# Single-Molecule Color-Stable Cool-WOLED Emitters with Multicolored Emission

**DOI:** 10.3390/molecules31071082

**Published:** 2026-03-26

**Authors:** Ming-Xing Song, Jinyu Wang, Zicong Pan, Yunkai Zhang, Lin Cui, Lixin Bao, Yuhao Wang, Ruiping Deng, Zhengkun Qin, Guangzhao Lu

**Affiliations:** 1 College of Information Technology, Jilin Engineering Research Center of Optoelectronic Materials and Devices, Jilin Normal University, Siping 136000, China; wangjyahh@163.com (J.W.); 13815095668@163.com (Z.P.); ykwillbebetter@163.com (Y.Z.); cuilin_1598@163.com (L.C.); 15943033509@163.com (L.B.); wangyh1110@163.com (Y.W.); 2 Jilin Provincial Key Laboratory of Wide Bandgap Semiconductor Material Growth and Device Applications, Jilin Normal University, Changchun 130103, China; 3 State Key Laboratory of Rare Earth Resource Utilization, Changchun Institute of Applied Chemistry, Chinese Academy of Sciences, Changchun 130022, China; dengrp@ciac.ac.cn; 4 Shenzhen Institute of Information Technology, Shenzhen 518172, China

**Keywords:** WOLEDs, single-molecular white-light, TADF, pure organic electroluminescent materials

## Abstract

Single-molecule white-light emitters have attracted much attention due to their potential applications in white organic light-emitting diodes (WOLEDs). Their key advantage lies in the ability to use a simple device structure, akin to that of monochromatic OLEDs, to produce WOLEDs. This approach not only simplifies the fabrication process but also reduces costs, improves device stability, and provides a shortcut for the rapid commercialization of WOLEDs. In this study, two novel single-molecule white-light emitters, SRFR-1PTZ (10-(4′-(9H-9,9′-spirobi[fluoren]-2-yl)-4a,10a-dihydro-10H-phenothiazine) and SRFR-2PTZ (2,7-bis(4a,10a-dihydro-10H-phenothiazin-10-yl)-9,9′-spirobi[fluorene]), were designed and synthesized, and successfully implemented in WOLED devices. Comprehensive photophysical characterization revealed that both compounds exhibited dual-emission characteristics in dichloromethane solution, displaying simultaneous fluorescence and phosphorescence. Notably, thermally activated delayed fluorescence (TADF) was clearly observed for SRFR-1PTZ, whereas SRFR-2PTZ did not exhibit TADF behavior. Electroluminescence studies demonstrated that both SRFR-1PTZ and SRFR-2PTZ served as good color-stable cool-white-light emitters under driving voltages of 7–10 V.

## 1. Introduction

White organic light-emitting diodes (WOLEDs) have emerged as a promising technology for next-generation display and lighting applications, owing to their superior characteristics that include high luminous efficiency, low power consumption, and rapid response time. These advantages have driven significant research interest in WOLEDs for both flat-panel displays and solid-state lighting systems [1,2,3,4,5,6]. In general, WOLEDs have been fabricated by encapsulating different emitters, such as the green–orange (GO, two elements doping) or red–green–blue (RGB, three elements doping) components, in a single emitting layer (EML) of the diodes, or by designing a multi-EML with different single color layers to obtain a WOLED [7,8,9,10,11,12,13,14,15,16]. On the one hand, achieving high color quality in such elaborate devices was challenging; on the other hand, device complexity introduced disadvantages such as high-cost, unstable devices structure, and difficulty in controlling charge transfer [17,18,19,20,21,22,23,24]. These challenges highlighted the importance of developing novel single-molecule white-light emitters capable of intrinsic GO dual-emission or RGB triple-emission. Such materials could serve as the sole active component in simplified EML structures, potentially overcoming the limitations of conventional multi-emitter systems while maintaining excellent electroluminescent performance.

In recent years, a considerable amount of research has been reported on single-molecule white-light emitters, including supramolecular assemblies, rare-earth complexes, transition metal coordination compounds, and thermally activated delayed fluorescence (TADF) materials. Among these, TADF materials are currently regarded as the most promising organic electroluminescent materials for commercial promotion due to their low cost and excellent luminescent characteristics. However, most studies have focused on warm white-light WOLEDs [16,25,26,27,28,29,30,31,32,33,34]. In contrast, cool-white-light (color temperature *CCT* > 4000 K) WOLEDs, which could suppress melatonin secretion and enhance alertness, were essential for various environments such as offices, classrooms, shopping malls, and hospitals [35]. Color stability was another important aspect, as it could affect human vision and eye health, and its evaluation was crucial for the recognition of potentially applicable WOLEDs, since a shift in the CIE (Commission Internationale de L’Eclairage) 1931 coordinates with the driving voltage or current at room temperature was often observed [13].

In order to find color-stable and cool-white emitters, two organic compounds, SRFR-1PTZ and SRFR-2PTZ (SRFR = 9,9′-spirobi [fluorene], PTZ = Phenothiazine), were designed and synthesized in this work. Specifically, building upon the design concept of SRFR-2PTZ, which had been reported in the literature as a host material for light-emitting devices, we designed SRFR-1PTZ, and also successfully synthesized SRFR-2PTZ [36,37]. The broad emission spectral bands of these compounds covered the entire visible light region (380–780 nm). This advantage arose from the sulfur atom-fused PTZ unit, which provided abundant frontier molecular orbitals to increase the probability of TADF occurring during the thermal activation process [38,39]. The CIE 1931 coordinates of the WOLEDs based on the compounds achieved (0.31, 0.26) (under a driving voltage of 3 V) and (0.30, 0.41) (under a driving voltage of 7–8 V), respectively. Furthermore, the color temperature of the WOLEDs was greater than 4000 K, which belonged to the standard range of cool-white-light [40]. Notably, the WOLEDs SRFR-1PTZ and SRFR-2PTZ emitted cool-white-light. Additionally, with the increase in operating voltage from 7 to 10 V, the EL spectra shifted only slightly, matching their stable CIE 1931 coordinates (∆x = 0.01; ∆y = 0.01). This indicated great color stability [40]. To further verify the photophysical properties of the two molecules, we performed calculations using density functional theory (DFT) and time-dependent DFT (TDDFT) methods to determine the energy gaps between singlet and triplet states (∆*E*_ST_), spin–orbit coupling (SOC) matrix elements, and nonadiabatic coupling (NAC) matrix elements [41].

## 2. Results and Discussion

### 2.1. Molecular Synthesis and Electrochemical Properties

**Figure 1:**
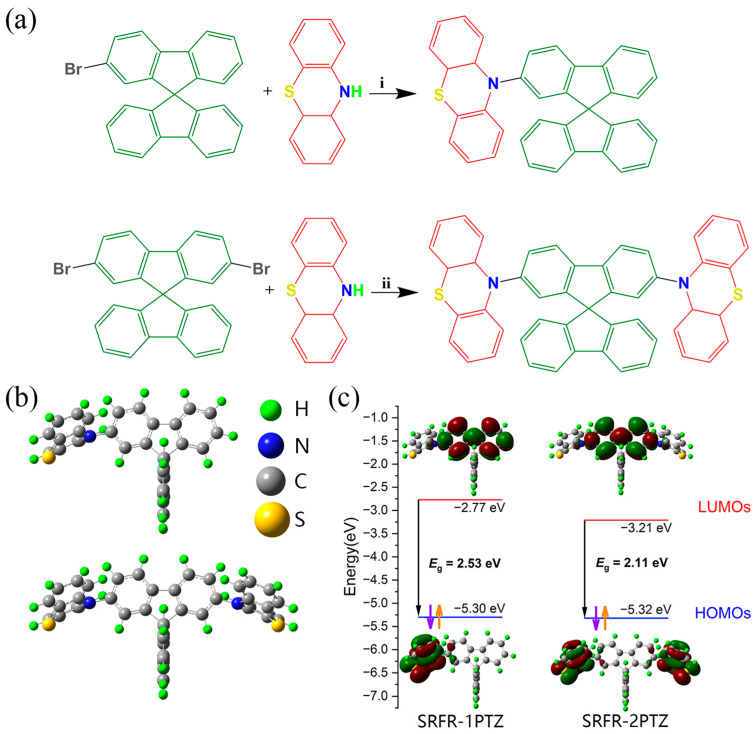
(**a**) Synthesis of compounds SRFR-1PTZ and SRFR-2PTZ; **i**: Synthesis of SRFR-1PTZ: A mixture of 2-bromo-9,9′-spirobi [9H-fluorene], phenothiazine (PTZ), P(t-Bu)_3_, NaOtBu, and Pd(OAc)_2_ in toluene was stirred under nitrogen at 90 °C for 12 h. Yield: 50.0%. **ii**: Synthesis of SRFR-2PTZ: A mixture of 2,7-dibromo-9,9′-spirobi [9H-fluorene], PTZ, P(t-Bu)_3_, NaOtBu, and Pd(OAc)_2_ in toluene was stirred under nitrogen at 90 °C for 18 h. Yield: 23.65%. (**b**) Optimized geometries of ground states of compounds under DFT level. (**c**) The energy levels, energy gaps, and HOMO and LUMO of the compounds obtained by DFT and TDDFT.

The target compounds SRFR-1PTZ and SRFR-2PTZ were successfully synthesized (Figure 1, the synthetic routes, the optimized geometries, and the molecular orbitals of the highest occupied molecular orbitals (HOMOs) and the lowest unoccupied molecular orbitals (LUMOs) based on the DFT). Similarly to Ir^III^ complexes (octahedron) that exhibited extremely impressive and comprehensive performance as phosphorescent emitters, the SRFR part presented a spatial three-dimensional structure (tetrahedral), which provided significant steric hindrance to reduce the phenomenon of quenching between molecules [42]. The HOMOs were localized on the SRFR group, while the LUMOs were localized on the PTZ part (Figure 1c and Tables S3 and S4, Supplementary Material). The process of charge transfer could be classified as through-bond charge transfer (TBCT), which accounted for the low photoluminescence quantum yields (PLQYs) of just 7.59% and 2.09% (shown in Table 1) [43,44,45,46,47,48].

The data of optimized geometries under DFT and TDDFT of ground states (S_g_), singlet excited states (S_e_), and triplet excited states (T_e_) for SRFR-1PTZ and SRFR-2PTZ are shown in Table S1 and Figure S1 (Supplementary Material). All the states of the compounds maintained a good symmetry, and the molecular structure distortion of the compounds between different states was small (<0.03 Å), as shown in the data of bond lengths. The differences in bond lengths and bond angles between the S_g_ and S_e_ were significant, especially for the dihedral angles C1-S-N-C3 and C3-N-C5-C6, which changed by 8–33 degrees. In contrast, the differences between S_e_ and T_e_ were relatively small (<5 degrees), which meant that the charge transition process between S_g_ and S_e_ required high energy barriers, while the energy barriers were smaller between S_e_ and T_e_ due to electron spin flipping. This was also one of the reasons for the low PLQYs of the compounds.

The purity and chemical structure of SRFR-1PTZ and SRFR-2PTZ were systematically characterized by ^1^H/^13^C-NMR spectra and mass spectra. The details are summarized in the Supplementary Material (Figures S2–S7, Supplementary Material). The results indicated that the molecular structures obtained experimentally were completely consistent with the theoretical design, and the products were pure substances.

The thermal properties of SRFR-1PTZ and SRFR-2PTZ were studied by thermogravimetric analysis (TGA) and differential scanning calorimetry (DSC) under the nitrogen atmosphere (Figures S8 and S9, Supplementary Material). For TGA, the degradation temperatures (T_d_, corresponding to a 5% weight loss) of SRFR-1PTZ and SRFR-2PTZ were determined to be 201.6 and 359.2 °C (Figure S9, Supplementary Material), respectively, and no discernible peaks were observed in DSC curves (Figure S8, Supplementary Material). The glass transition temperature (T_g_) of SRFR-1PTZ and SRFR-2PTZ was 127.7 and 98.0 °C, while the melting temperature (T_m_) was 266.5 and 335.3 °C, and the results indicated that SRFR-1PTZ and SRFR-2PTZ possessed exceptional thermal stability for device fabrication via evaporation methods and could withstand Joule heating during device operation.

### 2.2. Photophysical Properties

**Figure 2:**
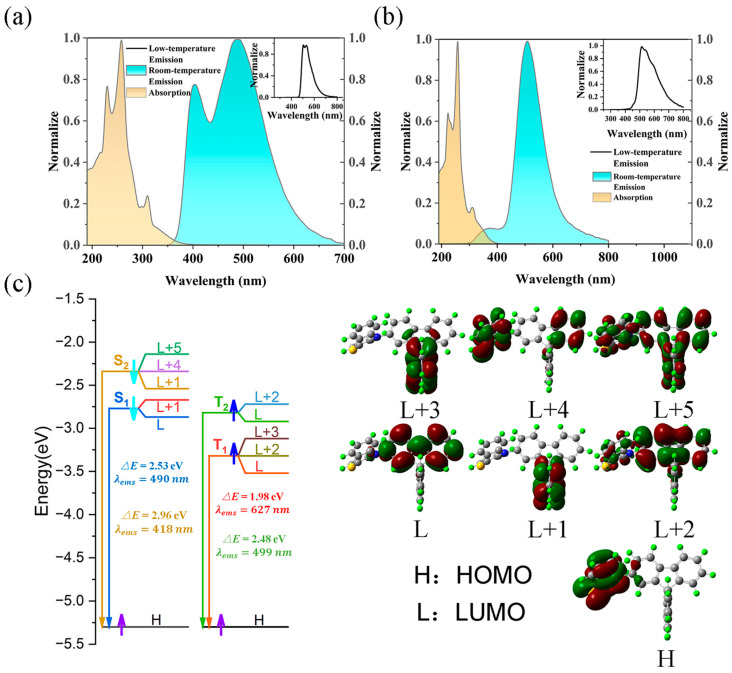
(**a**,**b**) The measured absorption spectra, room-temperature emission spectra, and low-temperature phosphorescence spectra (inset) of SRFR-1PTZ and SRFR-2PTZ. (**c**) Presentation of the energy levels, energy gaps, and orbital composition distribution of the activation orbitals joined in charge-transfer transitions for SRFR-1PTZ under DFT and TDDFT calculations.

The UV-Vis absorption spectra, room-temperature emission spectra, and low-temperature (77 K) phosphorescence emission spectra of SRFR-1PTZ and SRFR-2PTZ were measured in dichloromethane solution (Figure 2a,b). Some of the photophysical property parameters of the compounds are listed in Table 1. For ease of discussion, SRFR-1PTZ was used as an example for the analysis below. As shown in Figure 2a, the room-temperature emission spectrum exhibited two distinct emission peaks (404/488 nm), since the phenothiazine group adopted two conformations, which gave rise to fluorescence emission at different wavelength ranges of the phenothiazine group [49,50,51,52,53,54]. The full width at half maximum (FWHM) of the emission band of SRFR-1PTZ was measured to be 161 nm, indicating a broad spectral coverage. This value was slightly wider than that reported for a phenanthrocarbazole-based white emitter (PCTrPE, FWHM = 151 nm), which might be attributed to enhanced vibronic coupling and multiple conformational states of SRFR-1PTZ [55,56,57,58]. Furthermore, the spectrum nearly covered the entire visible region (350 nm–700 nm), indicating relatively weak light emission on the longer-wavelength side beyond 488 nm. The measured lifetime of SRFR-1PTZ was 5.57 μs (Table 1), suggesting the presence of long-life luminescence (fluorescence or TADF) in the emission spectrum. The low-temperature phosphorescence emission spectrum showed three peaks (506/531/621 nm). It is well-known that low-temperature phosphorescence spectroscopy primarily detects long-life luminescence (>ns). This observation further confirmed the presence of long-life luminescence in the photophysical processes of compound SRFR-1PTZ. Similarly, the emission spectrum of compound SRFR-2PTZ also contained long-lived luminescence, with an FWHM of 103 nm, reflecting a relatively narrower emission profile compared to SRFR-1PTZ. This bandwidth was comparable to solution-state values reported for phenylpyridinyl derivatives (62–68 nm), though measured in solid films where intermolecular interactions typically broaden emission spectra, suggesting reduced inhomogeneous broadening for SRFR-2PTZ [55,56,57,58]. The HOMO and LUMO energy levels obtained from cyclic voltammetry (CV) measurements (see Figures S19 and S20 in the Supplementary Material) and DFT/TDDFT calculations were −5.30/−2.77 eV for SRFR-1PTZ and −5.32/−3.21 eV for SRFR-2PTZ, respectively.

**Figure 3:**
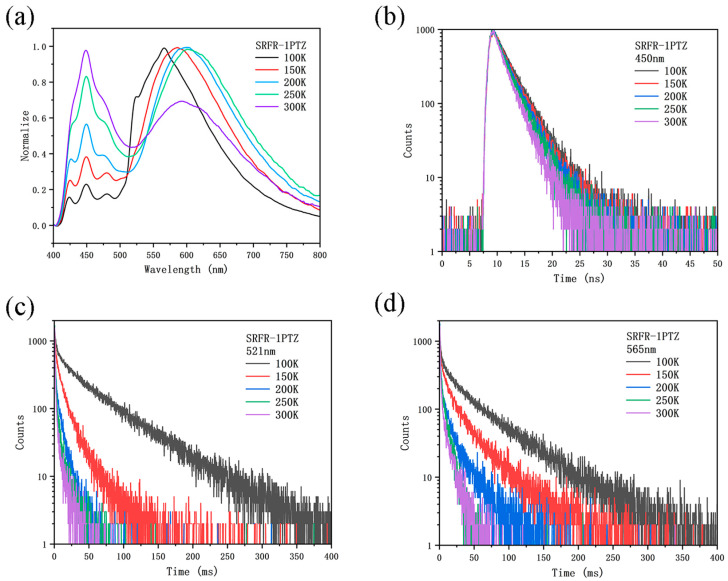
(**a**) Temperature-dependent luminescence spectra of SRFR-1PTZ as solid from 100 to 300 K. (**b**–**d**) Temperature-dependent transient PL decay curves of SRFR-1PTZ with 450 nm, 521nm, and 565 nm.

To further investigate the light-emission characteristics of the two molecules, temperature-dependent luminescence spectra of SRFR-1PTZ and SRFR-2PTZ in the solid state were recorded from 100 K to 300 K (Figure 3 and Figure S13). Compared with their spectra in CH_2_Cl_2_ solution, both compounds exhibited a pronounced red shift in the solid state, attributable to intermolecular close-packing effects. SRFR-1PTZ showed three emission peaks at 450 nm, 521 nm, and 565 nm, while SRFR-2PTZ displayed peaks at 450 nm, 475 nm, and 560 nm. Notably, the intensity of the 450 nm band for SRFR-1PTZ increased markedly with rising temperature, indicating the presence of thermally activated delayed fluorescence. Temperature-dependent transient PL decay curves provided further insight. The emissions at 450 nm for SRFR-1PTZ and at 450/475 nm for SRFR-2PTZ exhibited features characteristic of prompt fluorescence. In contrast, the longer-wavelength emissions (521/565 nm for SRFR-1PTZ and 560 nm for SRFR-2PTZ) were assigned to phosphorescence or TADF. Moreover, the lifetimes of these longer-wavelength components decreased significantly as the temperature increased from 100 K to 300 K. This behavior can be explained by the coexistence of two distinct rotamers in the rigid matrix, where restricted molecular rotation, together with enhanced non-radiative triplet decay (thermal quenching), leads to the observed lifetime reduction—a phenomenon previously reported for TADF materials [59,60]. The specific lifetime values for these wavelength components are listed in Table S7 (SRFR-1PTZ) and Table S8 (SRFR-2PTZ) in the Supplementary Material.

To further verify the above conclusions, the charge-transfer transitions accompanying the luminescence processes, ∆*E*_ST_, and the characteristics of the relevant excited-state molecular orbitals were calculated under TDDFT (Figure 2c), and the SOC matrix elements between singlet (S_x_, x = 1, 2, 3 …) and triplet (T_x_, x = 1, 2, 3 …) states (<T|H_SOC_|S>, Table S6, Supplementary Material) were also obtained. The calculated results showed that for SRFR-1PTZ, fluorescence emission at 490 nm corresponded to the transition from the singlet state S_1_ to the ground state S_0_ (S_1_→S_0_), which involved the charge-transfer transition between the HOMO, LUMO, and LUMO + 1 orbitals (LUMO→HOMO and LUMO + 1→HOMO), matching the experimental emission peak at 488 nm produced by one conformation of the compound, which belonged to TBCT and through-space charge-transfer (TSCT) transitions (Figure 2c) [61]. Similarly, the calculated emission peak at 418 nm corresponded to the S_2_→S_0_ transition, matching the experimental emission peak at 404 nm produced by the other conformation of the compound SRFR-1PTZ. Furthermore, the ∆*E*_ST_ values revealed that the reverse intersystem crossing (RISC) was very weak between the lowest triplet state (T_1_) and the lowest singlet excited state (S_1_) (∆*E*_S1T1_ = 0.55 eV), whereas the RISC occurred more readily between the second triplet state (T_2_) and S_1_ (∆*E*_S1T2_ = 0.05 eV). Furthermore, the NAC calculation yielded a value of <T_2_|H_NAC_|T_1_> = 0.31 (see Table S6 in the Supplementary Material), suggesting weak coupling between T_2_ and T_1_ states. In addition, during the RISC process between the T_2_ and S_1_ states, the charge-transfer transitions in the T_2_ state involved the L + 2 and L orbitals, whereas those in the S_1_ state involved the L and L + 1 orbitals. As illustrated in Figure 2c, the transitions between the L + 2 and L orbitals, as well as the local excitations within the L orbitals, exhibited distinct locally excited (LE) character. In contrast, the transitions between the L + 2 and L + 1 orbitals, as well as those between the L and L + 1 orbitals, demonstrated clear charge-transfer (CT) character. Therefore, the S_1_ state of SRFR-1PTZ exhibited hybrid local and charge-transfer (HLCT) characteristics—a phenomenon commonly reported in the literature [42,62,63]. All results mentioned above indicated that TADF emission did not occur between T_1_ and S_1_ but could occur between T_2_ and S_1_. The results of SOC matrix elements showed a large value between T_1_ and S_0_ (<T_1_|H_SOC_|S_0_> = 18.02), while it was small between T_2_ and S_0_ (<T_2_|H_SOC_|S_0_> = 0.2), indicating that the T_1_→S_0_ transition could produce phosphorescence emission, whereas phosphorescence emission from T_2_ to S_0_ was unlikely. Therefore, considering both the ∆*E*_ST_ and <T|H_SOC_|S> calculation results collectively, the emission spectrum of the compound SRFR-1PTZ was composed of fluorescence, TADF, and phosphorescence. To gain deeper insights into the kinetics of the excited-state processes, we further derived key rate constants from the calculated SOC and NAC matrix elements (Table S6, Supplementary Material). For SRFR-1PTZ, the RISC rate from T_2_ to S_1_ (K_RISC_ = 13.57 × 10^5^ s^−1^) slightly exceeded the internal conversion rate from T_2_ to T_1_ (K_nr_ = 12.18 × 10^5^ s^−1^), indicating that the T_2_→S_1_ RISC pathway could compete effectively with non-radiative decay, thereby enabling TADF. Furthermore, the phosphorescence radiative rate from T_1_ to S_0_ (K_r_ = 41.17 × 10^5^ s^−1^) was markedly larger than the RISC rate from T_1_ to S_1_ (K_RISC_ = 18.82 × 10^5^ s^−1^), implying that the T_1_ state preferentially decayed via phosphorescence rather than returning to S_1_. These kinetic data reinforced the conclusion that TADF in SRFR-1PTZ originated primarily from the T_2_→S_1_ channel, while phosphorescence emanated from T_1_→S_0_. Following the same analytical approach for SRFR-2PTZ, charge-transfer transitions between the first and second singlet states (S_1_ and S_2_) and the ground state (S_0_) were nearly absent, as indicated by the zero resonance intensities (*f*) in Table S5. Consequently, the fluorescence of SRFR-2PTZ primarily originated from high-energy excited states (S_3_, S_4_, and S_5_) to S_0_, which were inefficient for charge transfer. This inefficient charge-transfer pathway prevented the RISC process from occurring via the S_1_ or S_2_ states. Furthermore, the substantial energy gaps (∆*E*_S3T_ > 0.3 eV) between the triplet states (T_1_, T_2_, T_3_, and T_4_) and the S_3_ state precluded RISC through the S_3_ channel, consistent with established TADF principles [42]. The SOC matrix elements (<T|H_SOC_|S>, Table S6) confirmed the presence of phosphorescent radiation channels (T_1_, T_2_, T_3_, T_4_ → S_0_). Kinetic analysis based on the derived rate constants (Table S6, Supplementary Material) further elucidated the absence of TADF in SRFR-2PTZ. The internal conversion rate from T_2_ to T_1_ (K_nr_ = 46.27 × 10^5^ s^−1^) was substantially larger than the RISC rate from T_2_ to S_1_ (K_RISC_ = 16.72 × 10^5^ s^−1^), indicating that T_2_ excitons predominantly underwent non-radiative decay to T_1_ rather than reverting to S_1_. Moreover, the RISC rate from T_1_ to S_1_ was negligible (K_RISC_ = 0), whereas the phosphorescence radiative rate from T_1_ to S_0_ remained appreciable (K_r_ = 36.83 × 10^5^ s^−1^). These kinetic parameters collectively demonstrated that triplet excitons in SRFR-2PTZ were primarily dissipated through phosphorescence, with no efficient RISC pathway to support TADF. Therefore, SRFR-2PTZ exhibited only fluorescence and phosphorescence, without TADF.

Compared with SRFR-1PTZ, the phosphorescence contribution in SRFR-2PTZ was significantly enhanced, which could be attributed to the increased number of PTZ ligands in the molecule. As shown in Table S5 and Figure S12, a greater number of PTZ ligands led to more molecular orbitals participating in charge-transfer transitions, with increased orbital overlap localized on either the PTZ or SRFR moieties. This resulted in shorter effective charge-transfer distances and thus facilitated phosphorescent emission.

In summary, the combined photophysical and theoretical analyses confirmed that SRFR-1PTZ functioned as a TADF emitter, whereas SRFR-2PTZ did not exhibit TADF characteristics.

### 2.3. EL Performance

**Figure 4:**
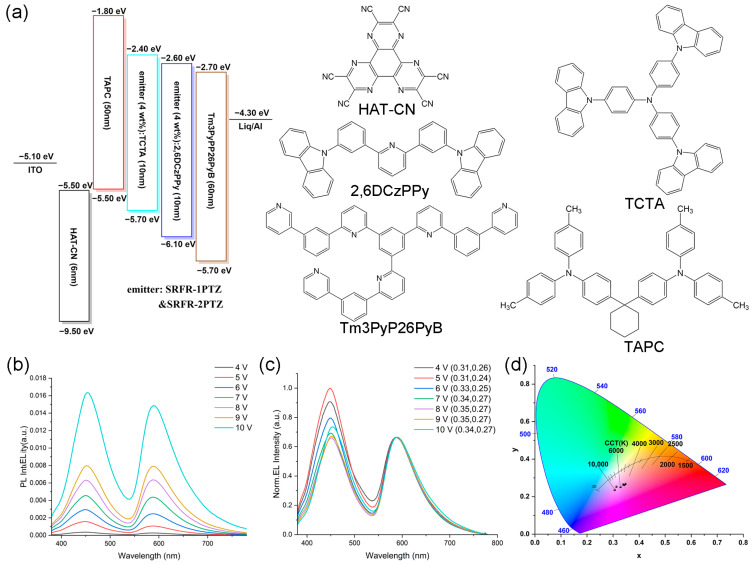
(**a**) Device structure for the compounds we obtained and the materials used in the device. (**b**,**c**) EL spectra under the driving voltages from 4 V to 10 V (Raw and Normalized) for SRFR-1PTZ. (**d**) CIE 1931 coordinates and color temperatures measured under the driving voltages from 4 V to 10 V for SRFR-1PTZ.

To verify the electroluminescent properties of the compounds, low-concentration doped OLEDs based on SRFR-1PTZ and SRFR-2PTZ as emitters were fabricated with the following device structure: ITO/HAT-CN (1,4,5,8,9,11-hexaazatriphenylene-hexacarbonitrile) (6 nm)/TAPC (di-[4-(N,N-ditolyl-amino)-phenyl]cyclohexane) (50 nm)/emitter (4 wt%): TCTA (4,4′,4″-tris(carbazol-9-yl)triphenylamine) (10 nm)/emitter (4 wt%): 2,6DCzPPy (2,6-bis(3-(9H-carbazol-9-yl)phenyl)pyridine) (10 nm)/Tm3PyP26PyB (1,3,5-tris(6-(3-(pyridin-3-yl)phenyl)pyridin-2-yl)benzene) (60 nm)/Liq (8-hydroxyquinolinolato-lithium) (2 nm)/Al (100 nm). This device configuration, which has been employed in our earlier studies, exhibited outstanding performance [64]. In this work, we carried over the device structure and fabrication procedure used in our previous studies (see Section S1.5 “Device fabrication and measurement” in the Supplementary Material for details). Figure 4a showed the energy-level diagram of the devices, as well as the molecular structure of the materials used. Obviously, the HOMO and LUMO energy levels (Table 1, Figure 4a) of the emitters lay within the HOMOs (−5.70 and −6.10 eV) and LUMOs (−2.40 and −2.60 eV) of the host materials (TCTA and 2,6DCzPPy), respectively. Therefore, good carrier trapping was expected in the device, suggesting that carrier trapping was the dominant electroluminescence (EL) mechanism. Furthermore, holes and electrons would be well-confined within the light-emitting layer.

The voltage-dependent electroluminescence emission spectra, the CIE 1931 coordinates, the correlated color temperatures, and the luminance–external quantum efficiency (L-EQE) curves of the two devices are illustrated in Figure 4b–d, Tables S9 and S10, and Figures S15–S18 (Supplementary Material). As shown in Figure 4, for SRFR-1PTZ, the EL spectrum exhibited two distinct peaks at 450 nm (sky-blue) and 590 nm (orange-red), which corresponded to the solid-state PL peaks of the compound. Together with the values of the CIE 1931 coordinates and the correlated color temperatures (*CCT* > 4000 K), these data confirmed that the SRFR-1PTZ-based device functioned as a cool-white OLED. The corresponding color rendering index (CRI) values are listed in Table S9 (Supplementary Material). Significantly, along with the operation voltage increasing from 7 to 10 V, the EL spectra of the WOLED changed slightly, consistent with their stable CIE 1931 coordinates (∆x = 0.01, ∆y = 0) (the complete voltage-dependent CIE 1931 data are listed in Table S9), which meant that the color stability of the material was considerable. This performance surpassed many reported systems, such as voltage-tunable devices based on phenylpyridinyl derivatives (ΔCIE > 0.05) and PCTrPE-based WOLEDs (Δx = 0.03, Δy = 0.02). The minimal chromaticity drift of our devices arose from the rigid spirobifluorene scaffold and stable phenothiazine-based charge-transfer characteristics, which effectively prevented electric-field-induced spectral shifts [55,56,57,58]. The corresponding full width at half-maximum (FWHM) values of the EL spectra under different driving voltages are summarized in Table S11 (Supplementary Material). Similarly, SRFR-2PTZ also displayed cool-white emission with good color stability: its EL spectra, CIE 1931 coordinates (∆x = 0.01, ∆y = 0.01; see Table S10 for full data) under driving voltages of 7–10 V, and color temperature (*CCT* > 6000 K) all confirmed a stable cool-white-light character. The CRI values for SRFR-2PTZ are provided in Table S10 (Supplementary Material). The external quantum efficiency (EQE) characteristics of both devices are summarized in Tables S9 and S10 (Supplementary Material). For SRFR-1PTZ, the EQE peaked at 0.362% under the initial driving voltage of 4 V and gradually decreased upon increasing the voltage, reaching 0.248% at 10 V. Notably, a more pronounced efficiency roll-off was observed above 7 V, indicating reduced stability under higher driving conditions. In contrast, SRFR-2PTZ exhibited a higher initial EQE of 1.004% at 4 V, which also declined with increasing voltage, falling to 0.370% at 10 V. Although both compounds showed typical efficiency roll-off behavior, SRFR-2PTZ maintained relatively better efficiency retention across the tested voltage range.

## 3. Materials and Methods

### 3.1. Experimental Section

All reagents and solvents were used as received from commercial suppliers. The target compounds SRFR-1PTZ and SRFR-2PTZ were synthesized via palladium-catalyzed C–N coupling reactions under nitrogen atmosphere, followed by purification and structural confirmation using ^1^H/^13^C NMR spectroscopy and high-resolution mass spectrometry (HR-MS). Thermal properties were evaluated by thermogravimetric analysis (TGA) and differential scanning calorimetry (DSC) under nitrogen. Photophysical measurements—including UV-Vis absorption, steady-state and time-resolved photoluminescence (PL), and low-temperature phosphorescence spectra—were performed in dichloromethane solution and solid state using commercial spectrophotometers and spectrofluorometers. Cyclic voltammetry (CV) was conducted in acetonitrile solution containing 0.1 M tetrabutylammonium hexafluorophosphate (n-Bu4NPF6) (after deoxygenation) as the supporting electrolyte. Organic light-emitting diodes (OLEDs) were fabricated by thermal evaporation in a high-vacuum chamber, and device performance (current–voltage–luminance characteristics, electroluminescence spectra, and external quantum efficiency) was measured in a nitrogen-filled glovebox using a spectroradiometer and source-measure unit.

### 3.2. Theoretical Calculations

DFT and TDDFT calculations were carried out with the Gaussian 16 package. Ground-state geometries were optimized at the B3LYP/def2-TZVP level. Excited-state energies, frontier molecular orbitals, SOC matrix elements, and NAC matrix elements were computed using DFT and TDDFT at the same level, with solvent effects (dichloromethane) included via the polarizable continuum model (PCM). The Multiwfn program was employed for orbital composition and charge-transfer analysis. To quantitatively compare the excited-state dynamics of SRFR-1PTZ and SRFR-2PTZ, key photophysical rate constants—including radiative (K_r_), non-radiative (K_nr_) and reverse intersystem crossing (K_RISC_) rate constants—were derived from temperature-dependent transient photoluminescence decay data.

Detailed experimental procedures, characterization data, computational parameters, calculation methods, and device fabrication steps are provided in the Supplementary Material.

## 4. Conclusions

In this work, two novel single-molecule emitters, SRFR-1PTZ and SRFR-2PTZ, were successfully developed, both exhibiting color-stable cool-white electroluminescence in simplified WOLEDs. SRFR-1PTZ demonstrated a combined emission mechanism involving fluorescence, phosphorescence, and TADF, while SRFR-2PTZ operated via fluorescence and phosphorescence only. The excellent color stability (∆CIE < 0.01) over a practical driving voltage range (7–10 V), attributed to the rigid molecular design, highlighted their significant potential for cost-effective and stable single-layer white-light devices. This work established a “rigid scaffold-enabled color stability” strategy, which provided a valuable design principle for the future development of efficient and color-pure single-molecule white emitters.

## Figures and Tables

**Table 1:** Photophysical properties of compounds SRFR-1PTZ and SRFR-2PTZ.

Compounds	*λ_rem_* ^(a)^ [nm]	*λ_lem_* ^(b)^ [nm]	*τ* ^(c)^ [μs]	CIE_r_ ^(d)^ (x,y)	CIE_l_ ^(e)^ (x,y)	PLQY ^(f)^ [%]	HOMO ^(g)^ [eV]	LUMO ^(h)^ [eV]
SRFR-1PTZ	404/488	506/531/621	5.57	(0.21, 0.30)	(0.32, 0.58)	7.59	−5.30	−2.77
SRFR-2PTZ	373/507	515/540/598	1.56	(0.26, 0.45)	(0.38, 0.52)	2.09	−5.32	−3.21

^(a)^ *λ_rem_* and ^(b)^ *λ_lem_*: Measured emission main peaks at room-temperature and low-temperature in dichloromethane solution; ^(c)^ *τ*: Measured delayed fluorescence lifetime in dichloromethane solution; ^(d)^ CIE_r_ and ^(e)^ CIE_l_: Color coordinates of emission spectrum at room-temperature and low-temperature in dichloromethane solution; ^(f)^ PLQY: Measured photoluminescence quantum yields in dichloromethane solution; ^(g)^ HOMO and ^(h)^ LUMO: The HOMO and LUMO energies levels obtained from cyclic voltammetry and DFT/TDDFT calculations.

## Data Availability

The data presented in this study are available on request from the corresponding authors.

## Supplementary Materials

The following supporting information can be downloaded at https://www.mdpi.com/article/10.3390/molecules31071082/s1. Refs. [63,65,66,67,68] are cited in the Supplementary Materials.

## References

[1] Kido J., Kimura M., Nagai K. (1995). Multilayer white light-emitting organic electroluminescent device. Science.

[2] Sun Y., Giebink N.C., Kanno H., Ma B., Thompson M.E., Forrest S.R. (2006). Management of singlet and triplet excitons for efficient white organic light-emitting devices. Nature.

[3] Reineke S., Lindner F., Schwartz G., Seidler N., Walzer K., Lüssem B., Leo K. (2009). White organic light-emitting diodes with fluorescent tube efficiency. Nature.

[4] Tang C.W., VanSlyke S.A. (1987). Organic electroluminescent diodes. Appl. Phys. Lett..

[5] Hung L.S., Chen C.H. (2002). Recent progress of molecular organic electroluminescent materials and devices. Mater. Sci. Eng. R Rep..

[6] Reineke S. (2015). Complementary LED technologies. Nat. Mater..

[7] Su S.-J., Gonmori E., Sasabe H., Kido J. (2008). Highly efficient organic blue-and white-light-emitting devices having a carrier- and exciton-confining structure for reduced efficiency roll-off. Adv. Mater..

[8] Li Z., Li B., Wei X., Liu J., Wang R., Hu X., Liu G., Gao H., Zhang Y., Lee C.-S. (2019). High efficiency, high color rendering index white organic light-emitting diodes based on thermally activated delayed fluorescence materials. Appl. Phys. Lett..

[9] Wu Z., Liu Y., Yu L., Zhao C., Yang D., Qiao X., Chen J., Yang C., Kleemann H., Leo K. (2019). Strategic-tuning of radiative excitons for efficient and stable fluorescent white organic light-emitting diodes. Nat. Commun..

[10] Wu S.-F., Li S.-H., Wang Y.-K., Huang C.-C., Sun Q., Liang J.-J., Liao L.-S., Fung M.-K. (2017). Organic light-emitting diodes: White organic LED with a luminous efficacy exceeding 100 lm W^−1^ without light out-coupling enhancement techniques. Adv. Funct. Mater..

[11] Im W.B., George N., Kurzman J., Brinkley S., Mikhailovsky A., Hu J., Chmelka B.F., DenBaars S.P., Seshadri R. (2011). Efficient and color-tunable oxyfluoride solid solution phosphors for solid-state white lighting. Adv. Mater..

[12] Yang Y., Wang X., Liu B., Zhang Y., Lv X., Li J., Li S., Wei L., Zhang H., Zhang C. (2018). Dependence of emitting light for LEDs fabricated by YAG:Ce crystal wafer on wafer thickness. J. Lumin..

[13] Higuchi T., Nakanotani H., Adachi C. (2015). High-efficiency white organic light-emitting diodes based on a blue thermally activated delayed fluorescent emitter combined with green and red fluorescent emitters. Adv. Mater..

[14] Eom S.-H., Zheng Y., Wrzesniewski E., Lee J., Chopra N., So F., Xue J. (2009). White phosphorescent organic light-emitting devices with dual triple-doped emissive layers. Appl. Phys. Lett..

[15] Xiang H., Wang R., Chen J., Li F., Zeng H. (2021). Research progress of full electroluminescent white light-emitting diodes based on a single emissive layer. Light Sci. Appl..

[16] Cole C.M., Yambem S.D. (2025). Thermally activated delayed fluorescent organic light emitting diodes: Solution processed to printed. Adv. Opt. Mater..

[17] Chen Z., Ho C.-L., Wang L., Wong W.-Y. (2020). Single-molecular white-light emitters and their potential WOLED applications. Adv. Mater..

[18] Mukherjee S., Thilagar P. (2014). Organic white-light emitting materials. Dyes Pigm..

[19] Bao L., Heagy M.D. (2014). A review of single white-light emitters: The quest for picture perfect dyes in the next generation of single layer WOLED displays. Curr. Org. Chem..

[20] Wang C., Dong H., Hu W., Liu Y., Zhu D. (2012). Semiconducting π-conjugated systems in field-effect transistors: A material odyssey of organic electronics. Chem. Rev..

[21] Chen Y.-H., Tang K.-C., Chen Y.-T., Shen J.-Y., Wu Y.-S., Liu S.-H., Lee C.-S., Chen C.-H., Lai T.-Y., Tung S.-H. (2016). Insight into the mechanism and outcoupling enhancement of excimer-associated white light generation. Chem. Sci..

[22] Pal K., Sharma V., Koner A.L. (2017). Single-component white-light emission via intramolecular electronic conjugation-truncation with perylenemonoimide. Chem. Commun..

[23] Wang K., Shi Y.-Z., Zheng C.-J., Liu W., Liang K., Li X., Zhang M., Lin H., Tao S.-L., Lee C.-S. (2018). Control of dual conformations: Developing thermally activated delayed fluorescence emitters for highly efficient single-emitter white organic light-emitting diodes. ACS Appl. Mater. Interfaces.

[24] Wu T.-L., Huang M.-J., Lin C.-C., Huang P.-Y., Chou T.-Y., Chen-Cheng R.-W., Lin H.-W., Liu R.-S., Cheng C.-H. (2018). Diboron compound-based organic light-emitting diodes with high efficiency and reduced efficiency roll-off. Nat. Photon..

[25] Li D., Wang J., Ma X. (2018). White-light-emitting materials constructed from supramolecular approaches. Adv. Opt. Mater..

[26] Zhang Q.-W., Li D., Li X., White P.B., Mecinović J., Ma X., Ågren H., Nolte R.J.M., Tian H. (2016). Multicolor photoluminescence including white-light emission by a single host–guest complex. J. Am. Chem. Soc..

[27] Liu D., Zhou Y.-N., Zhao J., Xu Y., Shen J., Wu M. (2017). An intensive green emitting terbium complex using a newly designed aromatic hyperbranched polyester as an efficient antenna ligand. J. Mater. Chem. C.

[28] Charytanowicz T., Sieklucka B., Chorazy S. (2023). Lanthanide hexacyanidoruthenate frameworks for multicolor to white-light emission realized by the combination of d-d, d-f, and f-f electronic transitions. Inorg. Chem..

[29] Sun W., Jiao S., Sun Y., Zhang D., Yang H., Zhou L. (2024). Highly efficient hybrid white organic light-emitting diodes with external quantum efficiency exceeding 30% by integrating electron-trapping sensitized structure and gradient-doping system. Adv. Funct. Mater..

[30] Yang G.-X., Chen Z., Yang Z., Liu D., Jiang S., Li D., Hu J., Li M., Su S.-J. (2024). Synergetic carbonyl and heptagonal structure for single-molecule white organic light-emitting diodes with dual thermally activated delayed fluorescence. Adv. Opt. Mater..

[31] Liu F., Liu H., Chen Y., He X., Cheng Z., Ma X., Qiao X., Ma D., Lu P. (2025). Achieving record high external quantum efficiency of 10.14% in nondoped single-emissive-layer WOLEDs utilizing hot exciton mechanism. CCS Chem..

[32] Dong B., Yan J., Li G., Xu Y., Zhao B., Chen L., Wang H., Li W. (2022). High luminance/efficiency monochrome and white organic light emitting diodes based pure exciplex emission. Org. Electron..

[33] Ivaniuk K., Stakhira P., Helzhynskyy I., Kutsiy S., Hotra Z., Deksnys T., Volyniuk D., Grazulevicius J.V., Gorbulic V. Contribution of fluorescence and exciplex emission into efficient white OLED. Proceedings of the 2020 IEEE 15th International Conference on Advanced Trends in Radioelectronics, Telecommunications and Computer Engineering (TCSET).

[34] Wu K., Liu D., Zhu L., Wu T., Xu Y., He C., Xiong Y., Zhao Z., Tang B.Z. (2025). Recent progress in triplet energy transfer systems toward organic afterglow materials. Commun. Chem..

[35] Lan B.-Y., Chuang C.-Y., Tseng C.-T., Ke J.-Y., Chu S.-Y., Kao P.-C. (2025). Improved performance of the thermally activated delayed fluorescence-based cool-white OLEDs with an efficient blue exciplex structure. Opt. Mater..

[36] Braveenth R., Bae I.-J., Han J.-H., Qiong W., Seon G., Raagulan K., Yang K., Park Y.H., Kim M., Chai K.Y. (2018). Utilizing a spiro core with acridine- and phenothiazine-based new hole transporting materials for highly efficient green phosphorescent organic light-emitting diodes. Molecules.

[37] Jesuraj P.J., Somasundaram S., Kamaraj E., Hafeez H., Lee C., Kim D., Won S.H., Shin S.T., Song M., Kim C.-S. (2020). Intramolecular charge transfer-based spirobifluorene-coupled heteroaromatic moieties as efficient hole transport layer and host in phosphorescent organic light-emitting diodes. Org. Electron..

[38] Tian X., Xiao S., Sun J., Yan J., Li G., Zhao B., Miao Y., Wang L., Wang H., Ma D. (2024). Dual fluorescence induced thermally activated delayed fluorescence materials based on 2,4-Diphenylthieno[3,2-d]pyrimidine with an efficient single-molecular white electroluminescence. Chem. Eng. J..

[39] Andruleviciene V., Leitonas K., Volyniuk D., Sini G., Grazulevicius J.V., Getautis V. (2021). TADF versus TTA emission mechanisms in acridan and carbazole-substituted dibenzo[a,c]phenazines: Towards triplet harvesting emitters and hosts. Chem. Eng. J..

[40] Ding D., Wang Z., Li C., Zhang J., Duan C., Wei Y., Xu H. (2020). Highly efficient and color-stable thermally activated delayed fluorescence white light-emitting diodes featured with single-doped single emissive layers. Adv. Mater..

[41] Nasiri S., Rabiei M., Shaki H., Hosseinnezhad M., Kalyani K., Palevicius A., Vilkauskas A., Janusas G., Nutalapati V., Kment S. (2024). What is TADF (thermally activated delayed fluorescence) compared to the mechanisms of FL (fluorescence), PH (phosphorescence), and TTA (triplet–triplet annihilation) based on a novel naphthalimide sulfonylphenyl derivative as a host?. J. Photochem. Photobiol. A Chem..

[42] Wong M.Y., Zysman-Colman E. (2017). Purely organic thermally activated delayed fluorescence materials for organic light-emitting diodes. Adv. Mater..

[43] Chen J.-X., Wang K., Zheng C.-J., Zhang M., Shi Y.-Z., Tao S.-L., Lin H., Liu W., Tao W.-W., Ou X.-M. (2018). Red organic light-emitting diode with external quantum efficiency beyond 20% based on a novel thermally activated delayed fluorescence emitter. Adv. Sci..

[44] Wang X., Wang S., Lv J., Shao S., Wang L., Jing X., Wang F. (2019). Through-space charge transfer hexaarylbenzene dendrimers with thermally activated delayed fluorescence and aggregation-induced emission for efficient solution-processed OLEDs. Chem. Sci..

[45] Shao S., Hu J., Wang X., Wang L., Jing X., Wang F. (2017). Blue thermally activated delayed fluorescence polymers with nonconjugated backbone and through-space charge transfer effect. J. Am. Chem. Soc..

[46] Rajamalli P., Senthilkumar N., Gandeepan P., Huang P.-Y., Huang M.-J., Ren-Wu C.-Z., Yang C.-Y., Chiu M.-J., Chu L.-K., Lin H.-W. (2016). A new molecular design based on thermally activated delayed fluorescence for highly efficient organic light emitting diodes. J. Am. Chem. Soc..

[47] Tsujimoto H., Ha D.-G., Markopoulos G., Chae H.S., Baldo M.A., Swager T.M. (2017). Thermally activated delayed fluorescence and aggregation induced emission with through-space charge transfer. J. Am. Chem. Soc..

[48] Ye J.-T., Wang L., Wang H.-Q., Pan X.-M., Xie H.-M., Qiu Y.-Q. (2018). Effective impact of dielectric constant on thermally activated delayed fluorescence and nonlinear optical properties: Through-bond/-space charge transfer architectures. J. Phys. Chem. C.

[49] Li B., Li Z., Guo F., Song J., Jiang X., Wang Y., Gao S., Wang J., Pang X., Zhao L. (2020). Realizing efficient single organic molecular white light-emitting diodes from conformational isomerization of quinazoline-based emitters. ACS Appl. Mater. Interfaces.

[50] Etherington M.K., Franchello F., Gibson J., Northey T., Santos J., Ward J.S., Higginbotham H.F., Data P., Kurowska A., Dos Santos P.L. (2017). Regio- and conformational isomerization critical to design of efficient thermally-activated delayed fluorescence emitters. Nat. Commun..

[51] Gibson J., Monkman A.P., Penfold T.J. (2016). The importance of vibronic coupling for efficient reverse intersystem crossing in thermally activated delayed fluorescence molecules. ChemPhysChem.

[52] Stockmann A., Kurzawa J., Fritz N., Acar N., Schneider S., Daub J., Engl R., Clark T. (2002). Conformational control of photoinduced charge separation within phenothiazine−pyrene dyads. J. Phys. Chem. A.

[53] Acar N., Kurzawa J., Fritz N., Stockmann A., Roman C., Schneider S., Clark T. (2003). Phenothiazine–pyrene dyads: Photoinduced charge separation and structural relaxation in the CT state. J. Phys. Chem. A.

[54] Tanaka H., Shizu K., Nakanotani H., Adachi C. (2014). Dual intramolecular charge-transfer fluorescence derived from a phenothiazine-triphenyltriazine derivative. J. Phys. Chem. C.

[55] Sharma R., Volyniuk D., Popli C., Bezvikonnyi O., Grazulevicius J.V., Misra R. (2018). Strategy toward tuning emission of star-shaped tetraphenylethene-substituted truxenes for sky-blue and greenish-white organic light-emitting diodes. J. Phys. Chem. C.

[56] Macionis S., Gudeika D., Bezvikonnyi O., Melnykov S., Guminilovych L., Simokaitiene J., Sargsyan S., Keruckiene R., Volyniuk D., Stakhira P. (2024). Effects of variation in phenylpyridinyl and di-tert-butyl-carbazolyl substituents of benzene on the performance of the derivatives in colour-tuneable white and exciplex-based sky-blue light-emitting diodes. Mater. Adv..

[57] Chatsirisupachai J., Nalaoh P., Kaiyasuan C., Chasing P., Sudyoadsuk T., Promarak V. (2021). Unique dual fluorescence emission in the solid state from a small molecule based on phenanthrocarbazole with an AIE luminogen as a single-molecule white-light emissive material. Mater. Chem. Front..

[58] Sych G., Volyniuk D., Bezvikonnyi O., Lytvyn R., Grazulevicius J.V. (2019). Dual interface exciplex emission of quinoline and carbazole derivatives for simplified nondoped white OLEDs. J. Phys. Chem. C.

[59] Russegger A., Debruyne A.C., Berrio D.C., Fuchs S., Marzi J., Schenke-Layland K., Dmitriev R.I., Borisov S.M. (2023). Bright and photostable TADF-emitting zirconium(IV) pyridinedipyrrolide complexes: Efficient dyes for decay time-based temperature sensing and imaging. Adv. Opt. Mater..

[60] Zhang Q., Kuwabara H., Potscavage W.J., Huang S., Hatae Y., Shibata T., Adachi C. (2014). Anthraquinone-based intramolecular charge-transfer compounds: Computational molecular design, thermally activated delayed fluorescence, and highly efficient red electroluminescence. J. Am. Chem. Soc..

[61] Ma F., Cheng Y., Zheng Y., Ji H., Hasrat K., Qi Z. (2019). Rational design of thermally activated delayed fluorescence emitters with aggregation-induced emission employing combined charge transfer pathways for fabricating efficient non-doped OLEDs. J. Mater. Chem. C.

[62] Sun Y., Fu X.-F., Hou C.-L., Zhang D.-H., Hu J.-X., Lin F.-L., Meng L., Zhou L., Chen X.-L., Lu C.-Z. (2025). Multifunctional hot-exciton fluorophore enabling efficient non-doped blue electron-fluorescence with negligible efficiency roll-off and phosphorescent OLEDs with ultra-low power consumption. Adv. Opt. Mater..

[63] Baryshnikov G., Minaev B., Ågren H. (2017). Theory and calculation of the phosphorescence phenomenon. Chem. Rev..

[64] Lu G., Wu R., Li N., Wang X., Zhou L., Yang C. (2022). Naphthyridine-based iridium(III) complexes for green to red OLEDs with EQEs over 30% and low efficiency roll-off. J. Mater. Chem. C.

[65] Byeon S.Y., Kim J.H., Lee J.Y. (2017). CN-modified host materials for improved efficiency and lifetime in blue phosphorescent and thermally activated delayed fluorescent organic light-emitting diodes. ACS Appl. Mater. Interfaces.

[66] Choi S.H., Lee C.H., Adachi C., Lee S.Y. (2020). Highly effective nicotinonitrile-derivatives-based thermally activated delayed fluorescence emitter with asymmetric molecular architecture for high-performance organic light-emitting diodes. Dyes Pigm..

[67] Jiang P., Miao J., Cao X., Xia H., Pan K., Hua T., Lv X., Huang Z., Zou Y., Yang C. (2022). Quenching-resistant multiresonance TADF emitter realizes 40% external quantum efficiency in narrowband electroluminescence at high doping level. Adv. Mater..

[68] Serevičius T., Skaisgiris R., Kreiza G., Dodonova J., Kazlauskas K., Orentas E., Tumkevičius S., Juršėnas S. (2021). TADF parameters in the solid state: An easy way to draw wrong conclusions. J. Phys. Chem. A.

