# Supplementary material for: Single-Molecule Color-Stable Cool-WOLED Emitters with Multicolored Emission
Source: Molecules. 2026 Mar 26;31(7):1082. doi: 10.3390/molecules31071082 (PMC13074305; doi:10.3390/molecules31071082)
Supplement: Supplementary file 1 [file molecules-31-01082-s001.zip › molecules-4025411-supplementary.pdf]

Supplementary Material

Single-Molecule Color-Stable Cool-WOLED Emitters with Multicolored Emission

Ming-Xing Song [1,2,*], Jinyu Wang [1,2], Zicong Pan [1,2], Yunkai Zhang [1,2], Lin Cui [1,2], Lixin Bao [1,2], Yuhao Wang [1,2], Ruiping Deng [3], Zhengkun Qin [1,2,*] and Guangzhao Lu [4,*]

[1] College of Information Technology, Jilin Engineering Research Center of Optoelectronic Materials and Devices, Jilin Normal University, Siping 136000, China; wangjyahh@163.com (J.W.); 13815095668@163.com (Z.P.); ykwillbebetter@163.com (Y.Z.); cuilin_1598@163.com (L.C.); 15943033509@163.com (L.B.); wangyh1110@163.com (Y.W.)
[2] Jilin Provincial Key Laboratory of Wide Bandgap Semiconductor Material Growth and Device Applications, Jilin Normal University, Changchun 130103, China
[3] State Key Laboratory of Rare Earth Resource Utilization, Changchun Institute of Applied Chemistry, Chinese Academy of Sciences, Changchun 130022, China; dengrp@ciac.ac.cn
[4] Shenzhen Institute of Information Technology, Shenzhen 518172, China
* Correspondence: mxsong@jlnu.edu.cn (M.-X.S.); zhengkun_qin@jlnu.edu.cn (Z.Q.); lugz@sziit.edu.cn (G.L.)

Contents

1. Experimental and theoretical section.

2. Synthesis and characterizations.

3. Supplementary data.

Table S1. Main optimized geometry structural parameters of SRFR-1PTZ and SRFR-2PTZ for the ground, singlet and triplet excited states under DFT/B3LYP and TDDFT/B3LYP level, respectively.

Table S2. Calculated absorption of SRFR-1PTZ and SRFR-2PTZ in CH_2Cl_2 media under TDDFT/B3LYP level.

Table S3. The frontier molecular orbital composited by fragments in the ground state for SRFR-1PTZ under TDDFT/B3LYP.

Table S4. The frontier molecular orbital composited by fragments in the ground state for SRFR-2PTZ under TDDFT/B3LYP.

Table S5. The ground and excited state energies as well as the energy gaps of both SRFR-1PTZ and SRFR-2PTZ under TDDFT/B3LYP and DFT/B3LYP level.

Table S6. Calculated spin-orbit coupling (SOC) matrix elements between states for SRFR-1PTZ and SRFR-2PTZ under DFT and TDDFT level.

Table S7. The luminescence lifetimes of SRFR-1PTZ measured at three characteristic emission wavelengths (450 nm, 521 nm, and 565 nm) over an identical temperature series (100 K, 150 K, 200 K, 250 K, and 300 K).

Table S8. The luminescence lifetimes of SRFR-2PTZ measured at three characteristic emission wavelengths (450 nm, 475 nm, and 560 nm) over an identical temperature series (100 K, 150 K, 200 K, 250 K, and 300 K).

Table S9. Electroluminescence performance of the device based on SRFR-1PTZ.

Table S10. Electroluminescence performance of the device based on SRFR-2PTZ.

Table S11. FWHMs of EL spectra for devices based on SRFR-1PTZ and SRFR-2PTZ at various voltages.

Figure S1. The sketch structures of the complexes SRFR-1PTZ and SRFR-2PTZ together with the numbers.

Figure S2. ^{1}H NMR spectrum of compound SRFR-1PTZ in $CDCl_3$.

Figure S3. ^{13}C NMR spectrum of compound SRFR-1PTZ in $CDCl_3$.

Figure S4. ^{1}H NMR spectrum of compound SRFR-2PTZ in $CDCl_3$.

Figure S5. ^{13}C NMR spectrum of compound SRFR-2PTZ in CD_2Cl_2.

Figure S6. HR-MS spectrum of compound SRFR-1PTZ.

Figure S7. HR-MS spectrum of compound SRFR-2PTZ.

Figure S8. DSC of SRFR-1PTZ and SRFR-2PTZ recorded under nitrogen atmosphere.

Figure S9. TGA of SRFR-1PTZ and SRFR-2PTZ recorded under nitrogen atmosphere.

Figure S10. The transient photoluminescence lifetimes of SRFR-1PTZ and SRFR-2PTZ.

Figure S11. Presentation of the energy levels and the energy gaps for SRFR-2PTZ under DFT and TDDFT calculations.

Figure S12. Presentation of the orbital composition distribution of the activation orbitals joined in charge-transfer transitions for SRFR-2PTZ under DFT and TDDFT calculations.

Figure S13. a) Temperature-dependent luminescence spectra of SRFR-2PTZ as solid from 100 to 300 K. b), c) and d) Temperature-dependent transient PL decay curves of SRFR-2PTZ with 450 nm, 475 nm and 560 nm.

Figure S14. Spectral color coordinates of SRFR-1PTZ a) and SRFR-2PTZ b) complexes at room and low temperatures.

Figure S15. EL spectra under the driving voltages from 4 V to 10 V (Raw) for SRFR-2PTZ.

Figure S16. EL spectra under the driving voltages from 4 V to 10 V (Normalized) for SRFR-2PTZ.

Figure S17. CIE coordinates and color-temperatures measured under the driving voltages from 4 V to 10 V for SRFR-2PTZ.

Figure S18. The L-EQE curves of the two compounds.

Figure S19. Cyclic voltammetry (CV) measurement of compound SRFR-1PTZ.

Figure S20. Cyclic voltammetry (CV) measurement of compound SRFR-2PTZ.

4. References

1. Experimental and theoretical section.

1.1 Materials and methods.

All reagents were used as received from commercial sources without further purification unless otherwise mentioned. Solvents for chemical synthesis were purified according to the standard procedures. NMR spectra were recorded on a Bruker Avance NEO 500 spectrometer (^{1}H NMR: 500 MHz, ^{13}C NMR: 125 MHz) (Bruker Corporation, Billerica, MA, USA). For SRFR-1PTZ, both ^{1}H and ^{13}C NMR spectra were measured in deuterated chloroform ($CDCl_3$). For SRFR-2PTZ, the ^{1}H NMR spectrum was acquired in $CDCl_3$, while the ^{13}C NMR spectrum was obtained in deuterated dichloromethane (CD_2Cl_2). The high-resolution mass spectrometry (HR-MS) for the compounds was conducted HRMS. Brand: Waters, Model: Xevo G2-XS QTof (Waters Corporation, Milford, MA, USA).

1.2 Thermal and electrochemical characterization.

Thermogravimetric analysis (TGA) was undertaken using Fully automatic comprehensive thermal analyzer HQT4 Instrument (Shanghai HENVEN Industrial Co., Ltd., Shanghai, China) at a heating rate of 10 ℃/min from 50 ℃ to 800 ℃ under nitrogen flushing. Cyclic voltammetry (CV) measurements were performed on a CHIF660F electrochemical workstation (CH Instruments, Inc., Shanghai, China) using *n*-Bu_4NPF_6 (0.1 M) in acetonitrile as electrolyte solution and ferrocene as an internal reference at a scan rate of 0.1 V s^{-1}. The CV cell consisted of a glassy carbon electrode, a Pt wire counter electrode, and a standard Ag/AgCl reference electrode. The polymers were spin-coated on the working electrode for measurements. The redox potentials were calibrated with ferrocene as an internal standard. The highest occupied molecular orbital (HOMO) energy levels of the polymers were estimated by the equation: $E_{\mathrm{HOMO}} = -(4.80 + E_{\mathrm{onset}}^{\mathrm{ox}})$ eV. The lowest unoccupied molecular orbital (LUMO) energy levels of the polymers were calculated by $E_{\mathrm{LUMO}} = E_{\mathrm{HOMO}} + E_{\mathrm{g}}$, and E_{g} was the optical band gap of the polymers in film.

1.3 Photophysical characterization.

The UV-Vis absorption spectra were obtained on a Shimadzu UV-2600 spectrophotometer (Shimadzu Corporation, Kyoto, Japan). Phosphorescence spectra were measured on an Edinburgh Instruments FLS1000 at 77 K (Edinburgh Instruments Ltd., Livingston, United Kingdom). The steady-state photoluminescence (PL) measurements were performed in a FLS1000, and the time-resolved PL studies were done by time-correlated single-photon counting (TCSPC) method in single photon counting controller. An FLS1000 PL spectrometer, having a 400 W Xe lamp, μF-400s flashlamp, and a PMT-900 detector from Edinburgh Instruments, was used for the slower time scale experiments. The absolute quantum yields were measured using an integrating sphere

(using Xe-400 lamp and PMT-900 along with an integrating sphere detector).

1.4 Quantum chemical calculations.

Density functional theory (DFT) and time-dependent density functional theory (TD-DFT) calculations were performed using the Gaussian 16 program package (Revision C.01) (Gaussian, Inc., Wallingford, PA, USA). The core idea of DFT was to express the interaction energy between electrons as a function of electron density, thus avoiding the complexity of dealing with multi-electron wave functions. Time-dependent density functional theory was an extension of the DFT for the study of excited states and time-dependent electronic properties. The ground state geometries were optimized using DFT, B3LYP/def2tzvp method. The computations of the singlet and triplet transition energies were carried out using TD-DFT by B3LYP/def2tzvp method based on the optimized ground state geometry, and excited state analysis were performed on Multiwfn 3.8 (Beijing, China), which had been successfully predicted for the charge-transfer compound [1-3].

Spin-orbit coupling (SOC) Calculation Method: The $T_1 \rightarrow S_0$ phosphorescence was a spin-forbidden process as stated by spin conservation law (ΔS=0). This law, however, could be relaxed by spin-orbit coupling (SOC) of the T_1 state with states of the singlet manifold [4]. The oscillator strength $f(T_1 \rightarrow S_0)$ (and phosphorescence rate) resulting from the SOC of the T_1 state with singlet states was defined as [5]:

$$f(T_1 \rightarrow S_0) = \sum_n [|\frac{\langle T_1|H_{SO}|S_n\rangle}{E(S_n)-E(T_1)}|^2 \times f(S_n \leftrightarrow S_0)] \quad \text{(S1)}$$

Here, T_1 and $E(T_1)$ represented the lowest triplet excited state and its energy, respectively; S_n and $E(S_n)$ denoted the n-th singlet excited state and its energy, respectively; H_{SO} was the spin-orbit coupling operator; and $f(S_n \leftrightarrow S_0)$ represented the oscillator strength for the transition between the S_n state and the ground state S_0.

Considering complexes SRFR-1PTZ and SRFR-2PTZ, the excited singlet states in close energetic proximity to the T_n state were of $\pi\pi^*$ character and had non-vanishing oscillator strength $f(S_n \leftrightarrow S_0)$. Then, the SOC matrix element

$$\langle T_n | H_{SO} | S_n \rangle$$

appeared to be the main factor in formula 1 that largely defined what oscillator strength would be picked up by $T_n \rightarrow S_0$ transition.

Analysis of Rate Constants: The radiative rate constant (k_r), non-radiative rate constant (k_{nr}), intersystem crossing rate constant (k_{ISC}), and reverse intersystem crossing rate constant (k_{RISC}) were calculated from the

following equations:

$$k_{\mathrm{p}} = \frac{1}{\tau_{\mathrm{p}}} \qquad (S2)$$

$$k_{\mathrm{d}} = \frac{1}{\tau_{\mathrm{d}}} \qquad (S3)$$

$$k_{\mathrm{r}} = \Phi_{\mathrm{p}} k_{\mathrm{p}} + \Phi_{\mathrm{d}} k_{\mathrm{d}} \approx \Phi_{\mathrm{p}} k_{\mathrm{p}} \qquad (S4)$$

$$k_{\mathrm{nr}} = \frac{1 - \Phi_{\mathrm{PL}}}{\Phi_{\mathrm{PL}}} k_{\mathrm{r}} \qquad (S5)$$

$$k_{\mathrm{ISC}} = k_{\mathrm{p}} - k_{\mathrm{r}} - k_{\mathrm{nr}} \qquad (S6)$$

$$k_{\mathrm{RISC}} = \frac{(k_{\mathrm{p}} k_{\mathrm{d}} \Phi_{\mathrm{d}})}{(k_{\mathrm{ISC}} \Phi_{\mathrm{p}})} \qquad (S7)$$

Where τ_{p} and τ_{d} represented the prompt and decay fluorescence lifetime, which were determined from transient photoluminescence (PL) spectra. The k_{p} and k_{d} represented the decay rate constants for prompt and delayed fluorescence, respectively. Φ_{p} and Φ_{d} indicated prompt and delayed fluorescence components and could be distinguished from the total Φ_{PL} by comparing the integrated intensities of prompt and delayed components in the transient decay curve.

1.5 Device fabrication and measurement.

1.5.1 Functions of each layer and rationales for material selection.

The device adopted a dual-emissive-layer structure: ITO / HAT-CN (6 nm) / TAPC (50 nm) / emitter (4 wt%):TCTA (10 nm) / emitter (4 wt%):2,6DCzPPy (10 nm) / Tm3PyP26PyB (60 nm) / Liq (2 nm) / Al (100 nm), aiming to balance charge transport and achieve efficient energy transfer. The patterned ITO glass (sheet resistance 10 Ω/sq) served as the transparent conductive anode, which was rigorously cleaned and treated with UV-ozone to enhance its work function and adhesion. HAT-CN functioned as the hole injection layer by forming a dipole layer at the ITO interface, significantly reducing the hole injection barrier. TAPC was selected as the hole transport layer due to its high hole mobility and matched HOMO level with the anode, while its high LUMO level effectively blocked electrons to confine excitons. The first emissive layer employed TCTA as the host doped with 4 wt% emitter, utilizing its high triplet energy to prevent energy back-transfer and enabling hole trapping near the interface. The second emissive layer used the bipolar host material 2,6DCzPPy doped with 4 wt% emitter, which broadened the exciton recombination zone and improved charge balance. Tm3PyP26PyB acted as the electron transport layer with high electron mobility and a deep HOMO level, efficiently transporting electrons while blocked holes. Liq served as the electron injection layer to reduce the electron injection barrier from the Al cathode and also protected the underlying organic film during Al deposition. The Al cathode, thermally evaporated, provided stable electron injection and acted as a reflective

layer in the bottom-emitting device configuration.

1.5.2 Fabrication instruments and methods.

All functional layers were deposited in an ultra-high vacuum environment using a multi-source organic molecular beam deposition system. The vacuum system, equipped with a turbo-molecular pump, achieved a base pressure of 3×10^{-4} to 5×10^{-4} Pa prior to deposition. The deposition rates and thicknesses of each layer were monitored and controlled in real-time using a quartz crystal microbalance thickness monitor. Organic materials were evaporated from resistively heated quartz crucibles. The deposition rate of host materials was maintained at 0.8–1.0 Å/s, while the dopant materials were co-evaporated at rates of approximately 0.2–0.3 Å/s to achieve the target doping concentration of 4 wt%. The Liq layer was deposited at a rate of 0.1–0.2 Å/s. The Al cathode was evaporated from a heated tungsten boat at a higher rate of 8–10 Å/s, with a shadow mask used to define the pixelated areas. After deposition, the film thicknesses were cross-verified using a surface profilometer.

1.5.3 Measurement instruments and methods.

Device performances were characterized inside a nitrogen-filled glovebox (O_2 < 0.1 ppm, H_2O < 0.1 ppm). All devices were tested without encapsulation or outcoupling enhancement, and electroluminescence measurements were conducted under dark ambient conditions at room temperature. The current density–voltage–luminance (J–V–L) characteristics were measured using a Keithley 2400 source meter for voltage sweeping (0–11 V, step 0.25 V) (Keithley Instruments, Cleveland, OH, USA), while luminance was collected by a PR735 spectroradiometer with the probe placed 5 cm away at a 2° viewing angle (Photo Research Inc., Simi Valley, CA, USA). Electroluminescence (EL) spectra and chromaticity were recorded by scanning the PR735 from 380 to 780 nm with a resolution of 1 nm, automatically calculating the CIE coordinates, correlated color temperature, and color rendering index. The external quantum efficiency (EQE) was calculated using the spectral integration method:

$$EQE = \frac{\pi \cdot L}{J \cdot V \cdot K_m \cdot \int EL(\lambda) \cdot V(\lambda)\, d\lambda} \tag{S8}$$

where K_m = 683 lm/W and $V(\lambda)$ was the CIE photopic luminosity function.

2. Synthesis and characterizations.

Synthesis of Compound SRFR-1PTZ: A mixture of 2-bromo-9,9'-spirobi[9H-fluorene] (0.0395 g, 0.1 mmol), phenothiazine (PTZ, 0.0299 g, 0.15 mmol), tri-tert-butylphosphine ($P(t\text{-}Bu)_3$, 10 w/v% in toluene, 75 μL, 0.0371 mmol), sodium tert-butoxide (NaOtBu, 0.0577 g, 0.6 mmol), and palladium acetate ($Pd(OAc)_2$, 0.00337 g, 0.015 mmol) in toluene (10 mL) was stirred under a nitrogen atmosphere at 90 °C for 12 hours. After cooling to room temperature, the mixture was filtered to isolate the crude product. Purification afforded the target compound SRFR-1PTZ as a solid (25.68 mg, 50.0% yield), based on a theoretical yield of 51.365 mg. The structure was confirmed by ^1H NMR spectroscopy. ^1H NMR (400 MHz, $CDCl_3$) δ 8.07 (d, J = 8.0 Hz, 1H), 7.91 (d, J = 7.6 Hz, 1H), 7.81 (d, J = 7.6 Hz, 2H), 7.42 (t, J = 7.7 Hz, 2H), 7.35 (t, J = 7.4 Hz, 2H), 7.20 – 7.10 (m, 3H), 6.97 – 6.88 (m, 2H), 6.85 – 6.67 (m, 8H), 6.10 (d, J = 7.9 Hz, 2H).

Synthesis of Compound SRFR-2PTZ: A mixture of 2,7-dibromo-9,9'-spirobi[9H-fluorene] (0.1423 g, 0.3 mmol), phenothiazine (PTZ, 0.1375 g, 0.69 mmol), tri-tert-butylphosphine ($P(t\text{-}Bu)_3$, 10 w/v% in toluene, 225 μL, 0.111 mmol), sodium tert-butoxide (NaOtBu, 0.171 g, 1.78 mmol), and palladium acetate ($Pd(OAc)_2$, 0.0102 g, 0.0454 mmol) in toluene (10 mL) was stirred under a nitrogen atmosphere at 90 °C for 18 hours. After cooling to room temperature, the mixture was filtered to isolate the crude product. Purification afforded the target compound SRFR-2PTZ as a solid (50.42 mg, 23.65% yield), based on a theoretical yield of 213.27 mg. The structure was confirmed by ^1H NMR spectroscopy. ^1H NMR (400 MHz, $CDCl_3$) δ 8.05 (d, J = 7.9 Hz, 2H), 7.69 (d, J = 7.7 Hz, 2H), 7.38 (d, J = 7.7 Hz, 2H), 7.26 (t, J = 7.6 Hz, 2H), 7.06 (t, J = 7.6 Hz, 2H), 6.87 (d, J = 6.2 Hz, 4H), 6.82 (d, J = 7.7 Hz, 2H), 6.75 (s, 2H), 6.68 (t, J = 5.0 Hz, 8H), 6.03 (d, J = 7.4 Hz, 4H).

3. Supplementary data.

Table S1

Main optimized geometry structural parameters of SRFR-1PTZ and SRFR-2PTZ for the ground, singlet and triplet excited states under DFT/B3LYP and TDDFT/B3LYP level, respectively.

Compound	State	Bond Length (Å)					Bond Angle (degree)			Dihedral Angle (degree)	
		$R_{C1\text{-}S}$	$R_{C2\text{-}S}$	$R_{C3\text{-}N}$	$R_{C4\text{-}N}$	$R_{C5\text{-}N}$	∠1	∠2	∠3	∠4	∠5
SRFR-1PTZ	S_g	1.767	1.767	1.415	1.415	1.437	99.5	121.4	118.3	145.3	98.0
	S_e	1.744	1.744	1.391	1.391	1.462	102.8	123.7	118.1	178.5	90.0
	T_e	1.728	1.728	1.394	1.394	1.447	104.3	126.5	116.8	173.5	90.0
SRFR-2PTZ	S_g	1.767	1.767	1.415	1.415	1.436	99.5	121.4	118.3	145.2	98.0
	S_e	1.744	1.744	1.391	1.391	1.463	102.8	123.9	118.1	177.0	89.7
	T_e	1.743	1.743	1.391	1.391	1.460	102.8	123.9	118.0	172.8	90.2

∠1=Angle C1-S-C2, ∠2=Angle C3-N-C4, ∠3= Angle C3-N-C5, ∠4= Angle C1-S-N-C3, ∠5= Angle C3-N-C5-C6; S_g=Ground state, S_e=Singlet Excited state, T_e=Triplet Excited state.

The data in **Table S1** correspond to the values shown in **Figure S1**.

Table S2

Calculated absorption of SRFR-1PTZ and SRFR-2PTZ in CH_2Cl_2 media under TDDFT/B3LYP level.

Compound	State	Energy (nm/eV)	Oscillator	Main configurations	Assign
SRFR-1PTZ	13	318/3.90	0.444	HOMO-3→LUMO(67%)	π(SRFR)→π*(SRFR/PTZ)
	15	268/4.62	0.166	HOMO-3→LUMO+1(68%)	π(SRFR)→π*(SRFR)
	18	261/4.75	0.284	HOMO-2→LUMO+2(63%)	π(SRFR/PTZ)→π*(SRFR/PTZ)
	27	248/4.99	0.213	HOMO-2→LUMO+4(57%)	π(SRFR/PTZ)→π*(SRFR/PTZ)
	28	248/5.01	0.191	HOMO-4→LUMO(59%)	π(SRFR)→π*(SRFR/PTZ)
SRFR-2PTZ	11	313/3.96	0.113	HOMO-2→LUMO(67%)	π(SRFR)→π*(SRFR/PTZ)
	14	309/4.01	0.077	HOMO→LUMO+3(40%)	π(SRFR/PTZ)→π*(SRFR/PTZ)
				HOMO→LUMO+8(39%)	π(SRFR/PTZ)→π*(SRFR/PTZ)
	18	289/4.29	0.115	HOMO-2→LUMO+1(62%)	π(SRFR)→π*(SRFR/PTZ)
	24	284/4.36	0.692	HOMO-5→LUMO(67%)	π(SRFR/PTZ)→π*(SRFR/PTZ)
	28	272/4.55	0.135	HOMO-5→LUMO+2(58%)	π(SRFR/PTZ)→π*(SRFR/PTZ)

Table S3

The frontier molecular orbital composited by fragments in the ground state for SRFR-1PTZ under TDDFT/B3LYP.

MO	Energy	Contribution		Assign
		SRFR	PTZ	
HOMO-9	-7.659	0.86	0.14	π[(SRFR)+(PTZ)]
HOMO-8	-7.366	0.28	0.72	π[(SRFR)+(PTZ)]
HOMO-7	-7.260	0	0.98	π(PTZ)
HOMO-6	-7.134	0.79	0.21	π[(SRFR)+(PTZ)]
HOMO-5	-7.086	0.99	0	π(SRFR)
HOMO-4	-6.935	0.96	0	π(SRFR)
HOMO-3	-6.419	0.96	0	π(SRFR)
HOMO-2	-6.417	0.28	0.72	π[(SRFR)+(PTZ)]
HOMO-1	-6.142	0.98	0	π(SRFR)
HOMO	-5.302	0.40	0.60	π[(SRFR)+(PTZ)]
LUMO	-1.470	0.89	0.11	π*[(SRFR)+(PTZ)]
LUMO+1	-1.312	0.97	0	π*(SRFR)
LUMO+2	-1.128	0.67	0.33	π*[(SRFR)+(PTZ)]
LUMO+3	-0.917	0.94	0.06	π*[(SRFR)+(PTZ)]
LUMO+4	-0.833	0.20	0.80	π*[(SRFR)+(PTZ)]
LUMO+5	-0.664	0.71	0.29	π*[(SRFR)+(PTZ)]
LUMO+6	-0.564	0.39	0.61	π*[(SRFR)+(PTZ)]
LUMO+7	-0.379	0.46	0.54	π*[(SRFR)+(PTZ)]
LUMO+8	-0.093	0.89	0.11	π*[(SRFR)+(PTZ)]
LUMO+9	0.119	0.45	0.55	π*[(SRFR)+(PTZ)]

Table S4

The frontier molecular orbital composited by fragments in the ground state for SRFR-2PTZ under TDDFT/B3LYP.

MO	Energy	Contribution		Assign
		SRFR	PTZ	
HOMO-9	-7.273	0	0.96	π(PTZ)
HOMO-8	-7.188	0.54	0.46	π[(SRFR)+(PTZ)]
HOMO-7	-7.119	0.99	0	π(SRFR)
HOMO-6	-6.994	0.88	0.12	π[(SRFR)+(PTZ)]
HOMO-5	-6.506	0.91	0.09	π[(SRFR)+(PTZ)]
HOMO-4	-6.434	0.36	0.64	π[(SRFR)+(PTZ)]
HOMO-3	-6.429	0.21	0.79	π[(SRFR)+(PTZ)]
HOMO-2	-6.194	0.97	0	π(SRFR)
HOMO-1	-5.326	0.36	0.64	π[(SRFR)+(PTZ)]
HOMO	-5.318	0.44	0.56	π[(SRFR)+(PTZ)]
LUMO	-1.635	0.84	0.16	π*[(SRFR)+(PTZ)]
LUMO+1	-1.331	0.95	0.05	π*[(SRFR)+(PTZ)]
LUMO+2	-1.249	0.74	0.26	π*[(SRFR)+(PTZ)]
LUMO+3	-0.989	0.10	0.90	π*[(SRFR)+(PTZ)]
LUMO+4	-0.949	0.90	0.10	π*[(SRFR)+(PTZ)]
LUMO+5	-0.828	0.17	0.83	π*[(SRFR)+(PTZ)]
LUMO+6	-0.684	0.83	0.17	π*[(SRFR)+(PTZ)]
LUMO+7	-0.675	0.47	0.53	π*[(SRFR)+(PTZ)]
LUMO+8	-0.661	0.17	0.83	π*[(SRFR)+(PTZ)]
LUMO+9	-0.459	0.37	0.63	π*[(SRFR)+(PTZ)]

Table S5

The ground and excited state energies as well as the energy gaps of both SRFR-1PTZ and SRFR-2PTZ under TDDFT/B3LYP and DFT/B3LYP level.

Compound	State	Energy Level [eV]	ΔE [a] [eV]	ΔE [b] [nm]	Main configurations	f [c]	ΔE_{S1T} [d] [eV]	ΔE_{S2T} [e] [eV]	ΔE_{S3T} [f] [eV]
SRFR-1PTZ	S_0	-5.30							
	S_1	-2.77	2.53	490	LUMO→HOMO(64%)<br>LUMO+1→HOMO(29%)	0.0006			
	S_2	-2.34	2.96	418	LUMO+1→HOMO(11%)<br>LUMO+4→HOMO(67%)<br>LUMO+5→HOMO(18%)	0.0048			
	T_1	-3.32	1.98	627	LUMO→HOMO(12%)<br>LUMO+2→HOMO(52%)<br>LUMO+3→HOMO(44%)		0.55	0.98	
	T_2	-2.82	2.48	499	LUMO→HOMO(69%)<br>LUMO+2→HOMO(10%)		0.05	0.48	
SRFR-2PTZ	S_0	-5.32							
	S_1	-3.21	2.11	588	LUMO→HOMO(70%)	0			
	S_2	-2.69	2.63	471	LUMO→HOMO-1(70%)	0			
	S_3	-2.28	3.04	408	LUMO+2→HOMO(29%)<br>LUMO+4→HOMO(42%)<br>LUMO+5→HOMO(45%)<br>LUMO+6→HOMO(17%)	0.0052			
	S_4	-1.92	3.40	364	LUMO+2→HOMO(17%)<br>LUMO+4→HOMO(45%)<br>LUMO+5→HOMO(40%)<br>LUMO+6→HOMO(31%)	0.0107			
	S_5	-1.72	3.60	345	LUMO→HOMO-2(65%)<br>LUMO+8→HOMO(23%)	0.0802			
	T_1	-3.21	2.11	589	LUMO→HOMO(68%)<br>LUMO+3→HOMO(13%)<br>LUMO+4→HOMO(11%)		0.00	0.52	0.90
	T_2	-3.00	2.32	535	LUMO→HOMO(19%)<br>LUMO+2→HOMO(27%)<br>LUMO+4→HOMO(47%)<br>LUMO+5→HOMO(36%)<br>LUMO+6→HOMO(12%)		-0.21	0.31	0.72
	T_3	-2.75	2.57	483	LUMO→HOMO-5(50%)<br>LUMO→HOMO-2(43%)		-0.46	0.06	0.47
	T_4	-2.68	2.64	470	LUMO→HOMO-1(69%)		-0.35	-0.01	0.40

[a] ΔE ***and*** [b] ΔE: The energy gaps between S_0 and the low excited states; [c] f: The resonance intensity between S_0 and the low triplet excited states; [d] ΔE_{S1T}, [e] ΔE_{S2T} **and** [f] ΔE_{S3T}: The energy gaps between S_1, S_2 and S_3 and the low triplet excited states.

Table S6

Calculated spin-orbit coupling (SOC) matrix elements between states for SRFR-1PTZ and SRFR-2PTZ under DFT and TDDFT level.

Compound	States	MS [a]=0		MS=-1		MS=1		$\langle T\vert H_{SOC}\vert S\rangle$	$\langle T\vert H_{NAC}\vert S\rangle$ [d]	K_r [e]	K_{nr} [f]	K_{RISC} [g]
		Re [b]	Im [c]	Re	Im	Re	Im	(cm^{-1})	(cm^{-1})	[10^5 s^{-1}]	[10^5 s^{-1}]	[10^5 s^{-1}]
	$T_1\rightarrow S_0$	0.00	19.68	0.00	9.56	0.00	-9.56	23.88		41.17		
	$T_2\rightarrow S_0$	0.00	0.01	-0.15	0.00	-0.15	-0.00	0.21				
	$T_2\rightarrow S_1$	0.00	0.38	-0.00	-0.11	-0.00	0.11	0.41				13.57
SRFR-1PTZ	$T_1\rightarrow S_1$	0.00	-0.00	-0.57	-0.00	-0.57	0.00	0.81				18.82
	$S_2\rightarrow T_2$	0.00	0.02	0.00	-0.09	0.00	0.09	0.13				
	$S_2\rightarrow T_1$	0.00	0.00	-0.34	-0.00	-0.34	0.00	0.48				
	$T_2\rightarrow T_1$								0.31		12.18	
	$T_1\rightarrow S_0$	0.00	18.56	0.00	0.00	0.00	-0.00	18.56		36.83		
	$T_2\rightarrow S_0$	0.00	0.00	0.00	-14.35	0.00	14.35	20.29				
	$T_3\rightarrow S_0$	0.00	-17.96	-0.00	-0.00	-0.00	0.00	17.96				
	$T_4\rightarrow S_0$	0.00	-0.00	-0.00	11.22	-0.00	-11.22	15.87				
	$S_1\rightarrow T_4$	0.00	0.00	0.45	0.00	0.45	-0.00	0.64				
	$S_1\rightarrow T_3$	0.00	0.00	-0.00	-0.00	-0.00	0.00	0.00				
	$T_2\rightarrow S_1$	0.00	-0.00	-0.44	-0.00	-0.44	0.00	0.62				16.72
	$T_1\rightarrow S_1$	0.00	-0.00	0.00	0.00	0.00	-0.00	0.00				0.00
	$S_2\rightarrow T_4$	0.00	0.00	0.00	-0.00	0.00	0.00	0.00				
	$S_2\rightarrow T_3$	0.00	0.00	0.40	0.00	0.40	-0.00	0.57				
	$S_2\rightarrow T_2$	0.00	-0.00	-0.00	0.00	-0.00	-0.00	0.00				
SRFR-2PTZ	$S_2\rightarrow T_1$	0.00	-0.00	-0.47	-0.00	-0.47	0.00	0.66				
	$S_3\rightarrow T_4$	0.00	-0.00	0.12	0.00	0.12	-0.00	0.17				
	$S_3\rightarrow T_3$	0.00	0.00	-0.00	-0.00	-0.00	0.00	0.00				
	$S_3\rightarrow T_2$	0.00	-0.00	0.32	-0.00	0.32	0.00	0.45				
	$S_3\rightarrow T_1$	0.00	0.00	-0.00	0.00	-0.00	-0.00	0.00				
	$S_4\rightarrow T_4$	0.00	0.00	0.00	-0.00	0.00	0.00	0.00				
	$S_4\rightarrow T_3$	0.00	0.00	0.12	0.00	0.12	-0.00	0.17				
	$S_4\rightarrow T_2$	0.00	0.00	0.00	0.00	0.00	-0.00	0.00				
	$S_4\rightarrow T_1$	0.00	-0.00	0.34	0.00	0.34	-0.00	0.48				
	$S_5\rightarrow T_4$	0.00	-0.00	-0.00	-0.02	-0.00	0.02	0.03				
	$S_5\rightarrow T_3$	0.00	-0.14	-0.00	0.00	-0.00	-0.00	0.14				
	$S_5\rightarrow T_2$	0.00	0.00	0.00	0.01	0.00	-0.01	0.01				
	$S_5\rightarrow T_1$	0.00	0.04	0.00	-0.00	0.00	0.00	0.04				
	$T_2\rightarrow T_1$										46.27	

[a]: MS: Substates under spin magnetic quantum number in triple excited states. [b]: Re: Re usually represents the real part of the compound number in calculating. [c]:Im: Im usually represents the imaginary part of the compound number in calculating. [d]: $\langle T|H_{NAC}|S\rangle$: Nonadiabatic coupling matrix element between triplet states, given in cm^{-1}, describing the internal conversion process between triplet excited states. [e] K_r: Radiative rate constant. [f] K_{nr}: Non-radiative rate constant. [g] K_{RISC}: Reverse intersystem crossing rate constant.

Table S7

The luminescence lifetimes of SRFR-1PTZ measured at three characteristic emission wavelengths (450 nm, 521 nm, and 565 nm) over an identical temperature series (100 K, 150 K, 200 K, 250 K, and 300 K).

Wavelength / Lifetime	450 nm	521 nm	565 nm
$\tau_{100\,K}$	2.9906 ns	53.7186 ms	47.9811 ms
$\tau_{150\,K}$	2.8237 ns	14.0632 ms	25.8474 ms
$\tau_{200\,K}$	2.6254 ns	6.0615 ms	13.4294 ms
$\tau_{250\,K}$	2.4337 ns	4.4667 ms	5.8968 ms
$\tau_{300\,K}$	2.1609 ns	3.0118 ms	5.6238 ms

Table S8

The luminescence lifetimes of SRFR-2PTZ measured at three characteristic emission wavelengths (450 nm, 475 nm, and 560 nm) over an identical temperature series (100 K, 150 K, 200 K, 250 K, and 300 K).

Wavelength / Lifetime	450 nm	475 nm	560 nm
$\tau_{100\,K}$	2.3003 ns	2.3638 ns	27.5398 ms
$\tau_{150\,K}$	2.3115 ns	2.3424 ns	18.8725 ms
$\tau_{200\,K}$	2.2661 ns	2.3135 ns	6.0760 ms
$\tau_{250\,K}$	2.2203 ns	2.3031 ns	2.3334 ms
$\tau_{300\,K}$	2.1346 ns	2.2286 ns	2.2413 ms

Table S9

Electroluminescence performance of the device based on SRFR-1PTZ.

Current Density (mA/cm^2)	Voltage (V)	Luminance (cd/m^2)	CE (cd/A)	PE (lm/W)	EQE (%)	CIE (x,y)	CCT (K)	CRI
2.233	4	13.079	0.586	0.460	0.362	(0.31,0.26)	7649.2	77.48
10.153	5	49.847	0.491	0.308	0.319	(0.31,0.24)	9656.8	76.31
20.728	6	107.16	0.517	0.271	0.320	(0.33,0.25)	5929.5	75.65
35.426	7	183.23	0.517	0.232	0.311	(0.34,0.27)	4748.5	73.60
54.902	8	265.68	0.484	0.190	0.289	(0.35,0.27)	4453.9	73.61
78.171	9	336.63	0.431	0.150	0.259	(0.35,0.27)	4546.4	73.68
164.602	10	664.52	0.404	0.127	0.248	(0.34,0.27)	5103.9	74.84

Table S10

Electroluminescence performance of the device based on SRFR-2PTZ.

Current Density (mA/cm²)	Voltage (V)	Luminance (cd/m²)	CE (cd/A)	PE (lm/W)	EQE (%)	CIE (x,y)	CCT (K)	CRI
3.504	4	96.539	2.755	2.098	1.004	(0.27,0.47)	7035.8	74.82
9.142	5	215.33	2.355	1.495	0.893	(0.27,0.46)	7223.9	73.88
19.616	6	362.3	1.847	0.959	0.733	(0.28,0.44)	7241.0	73.80
35.406	7	540.29	1.526	0.670	0.631	(0.29,0.42)	7033.4	74.83
52.912	8	708.82	1.340	0.528	0.567	(0.29,0.41)	6815.8	75.92
90.034	9	993.58	1.104	0.382	0.475	(0.30,0.41)	6546.9	77.27
141.729	10	1213.5	0.856	0.264	0.370	(0.30,0.41)	6415.3	77.92

Table S11

FWHMs of EL spectra for devices based on SRFR-1PTZ and SRFR-2PTZ at various voltages.

Compound	Voltage (V)	FWHM (nm)
SRFR-1PTZ	4	87
	5	211
	6	83
	7	227
	8	146
	9	82
	10	222
SRFR-2PTZ	4	66
	5	66
	6	70
	7	76
	8	85
	9	96
	10	99

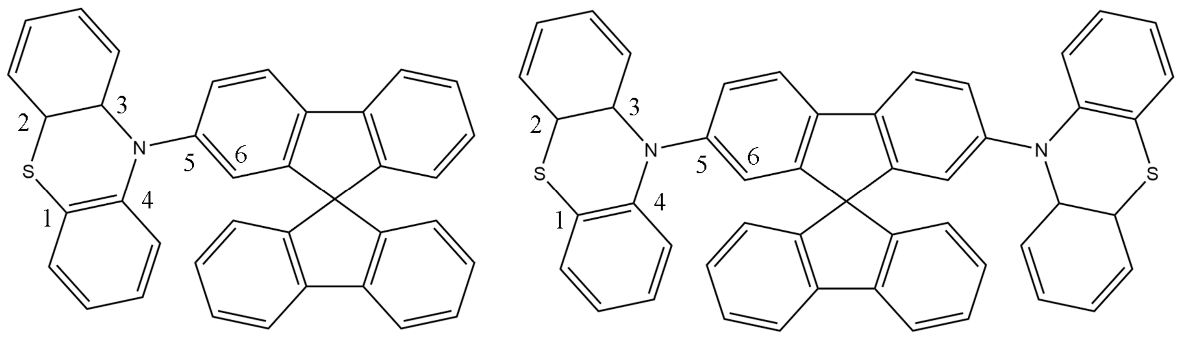

SRFR-1PTZ SRFR-2PTZ

Figure S1. The sketch structures of the complexes SRFR-1PTZ and SRFR-2PTZ together with the numbers.

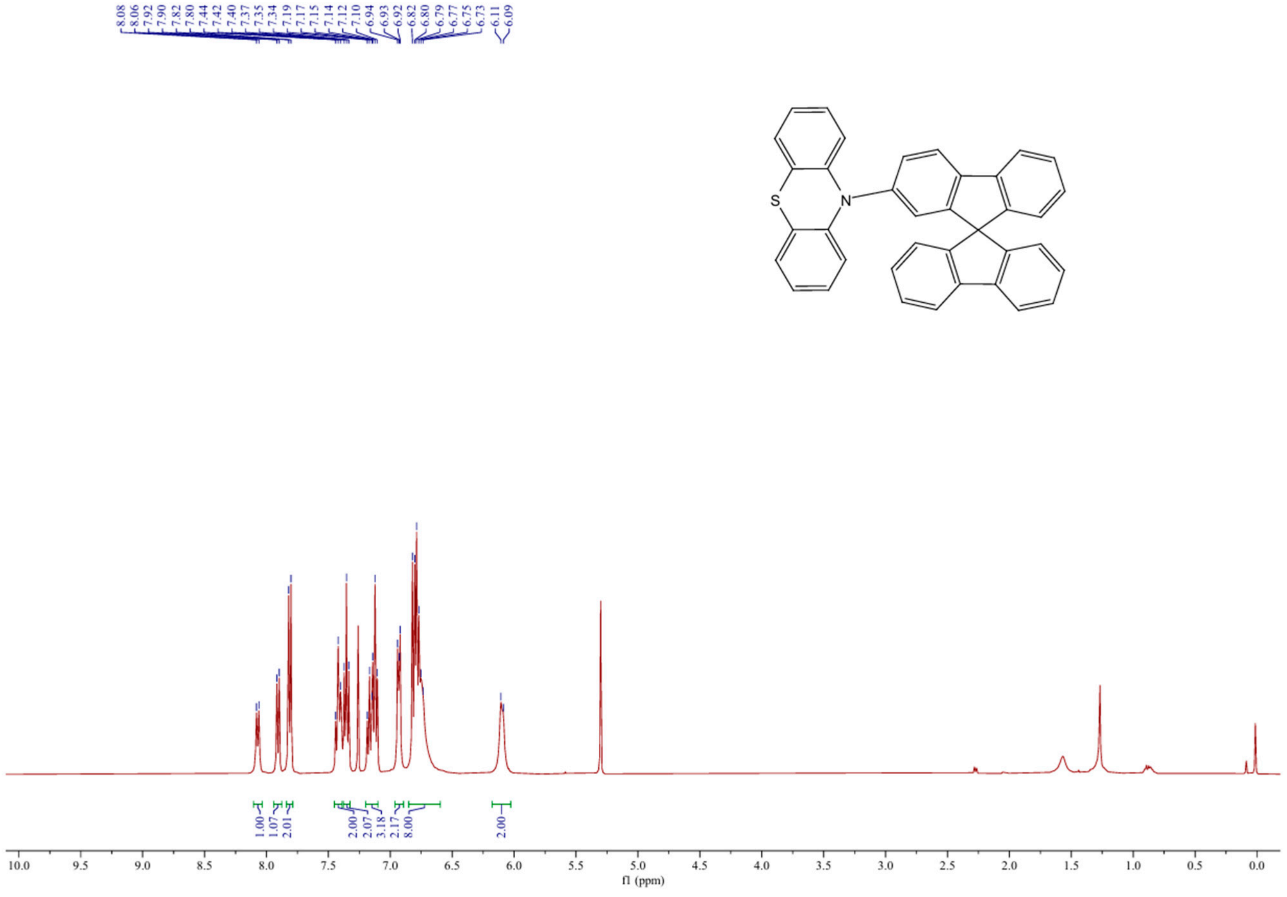

Figure S2. ^{1}H NMR spectrum of compound SRFR-1PTZ in $CDCl_3$.

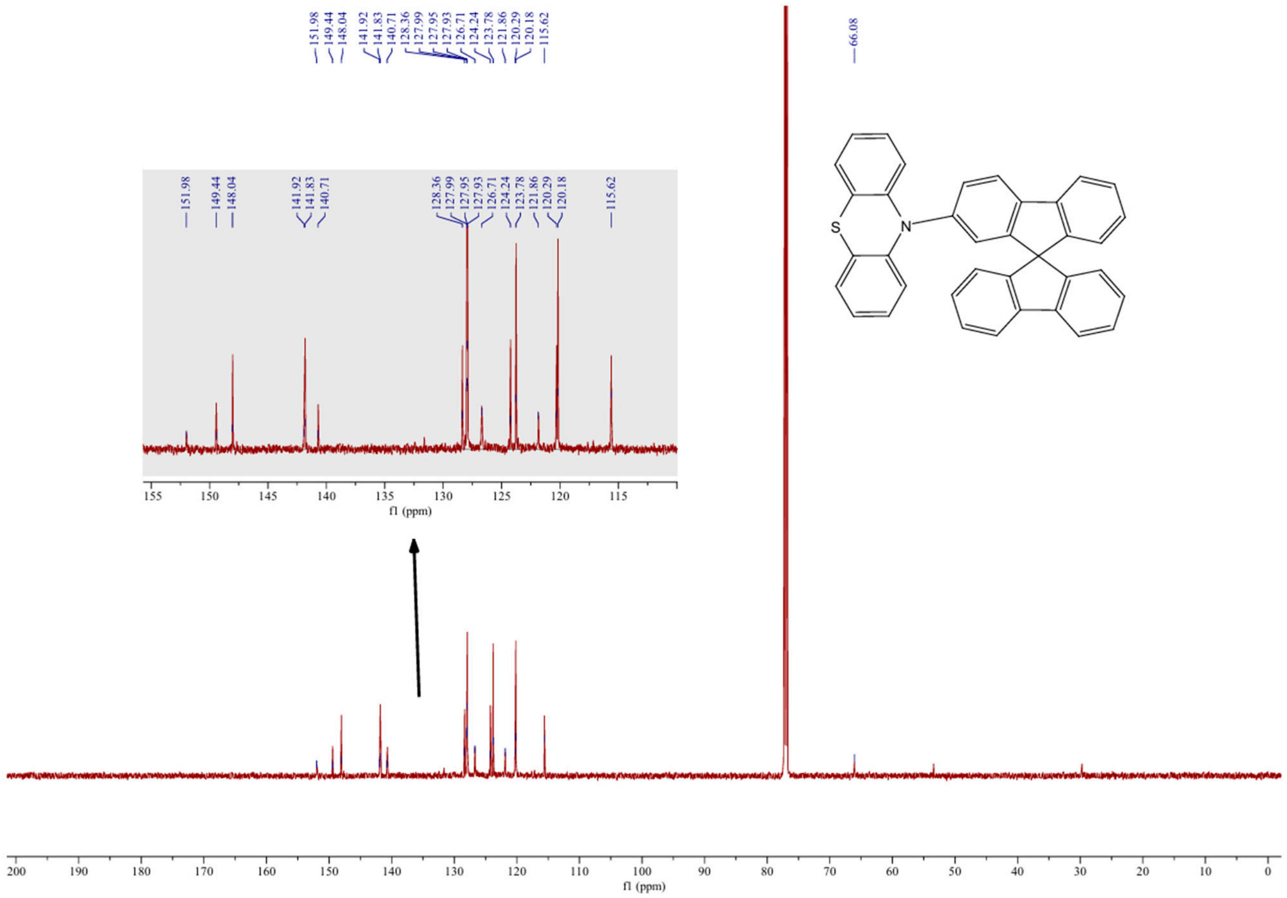

Figure S3. ^{13}C NMR spectrum of compound SRFR-1PTZ in $CDCl_3$.

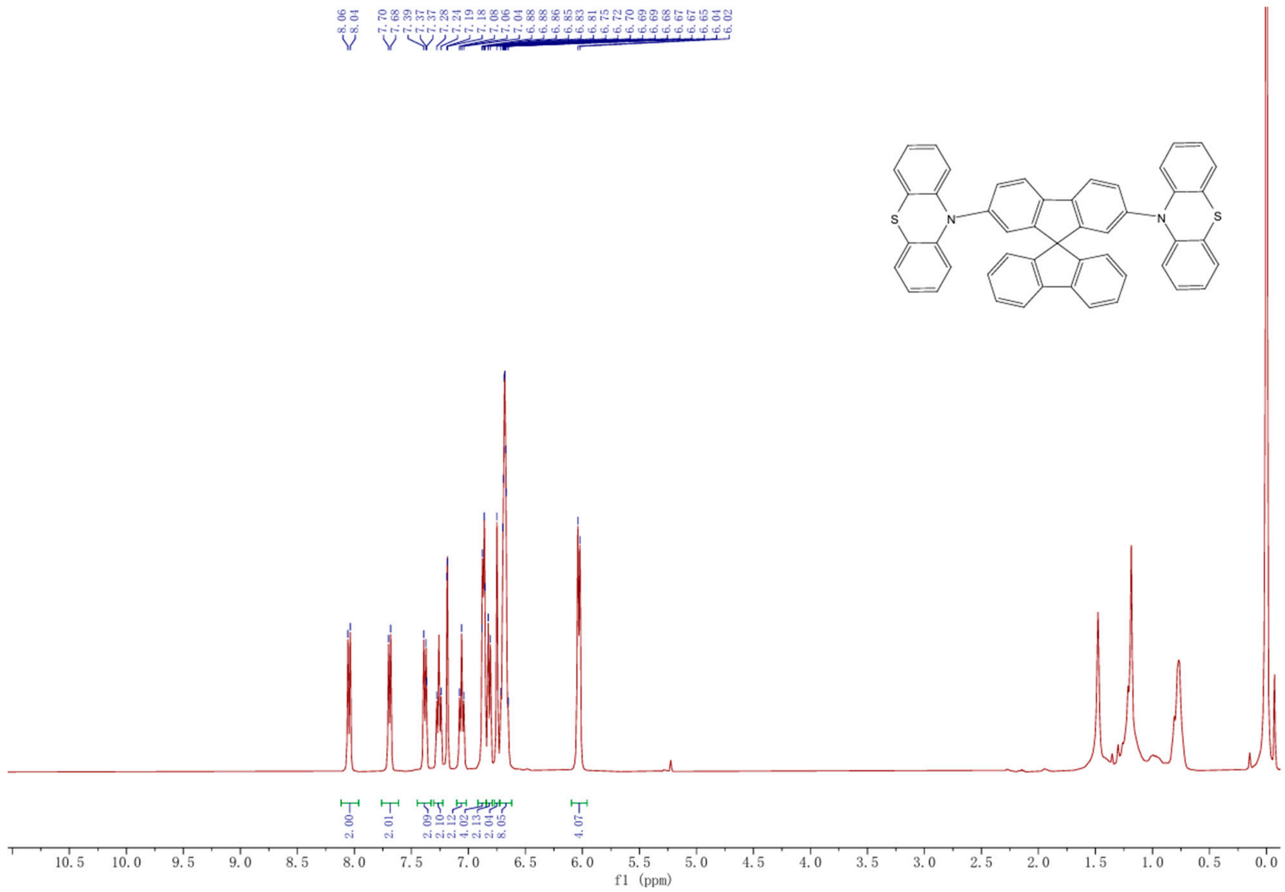

Figure S4. ^{1}H NMR spectrum of compound SRFR-2PTZ in $CDCl_3$.

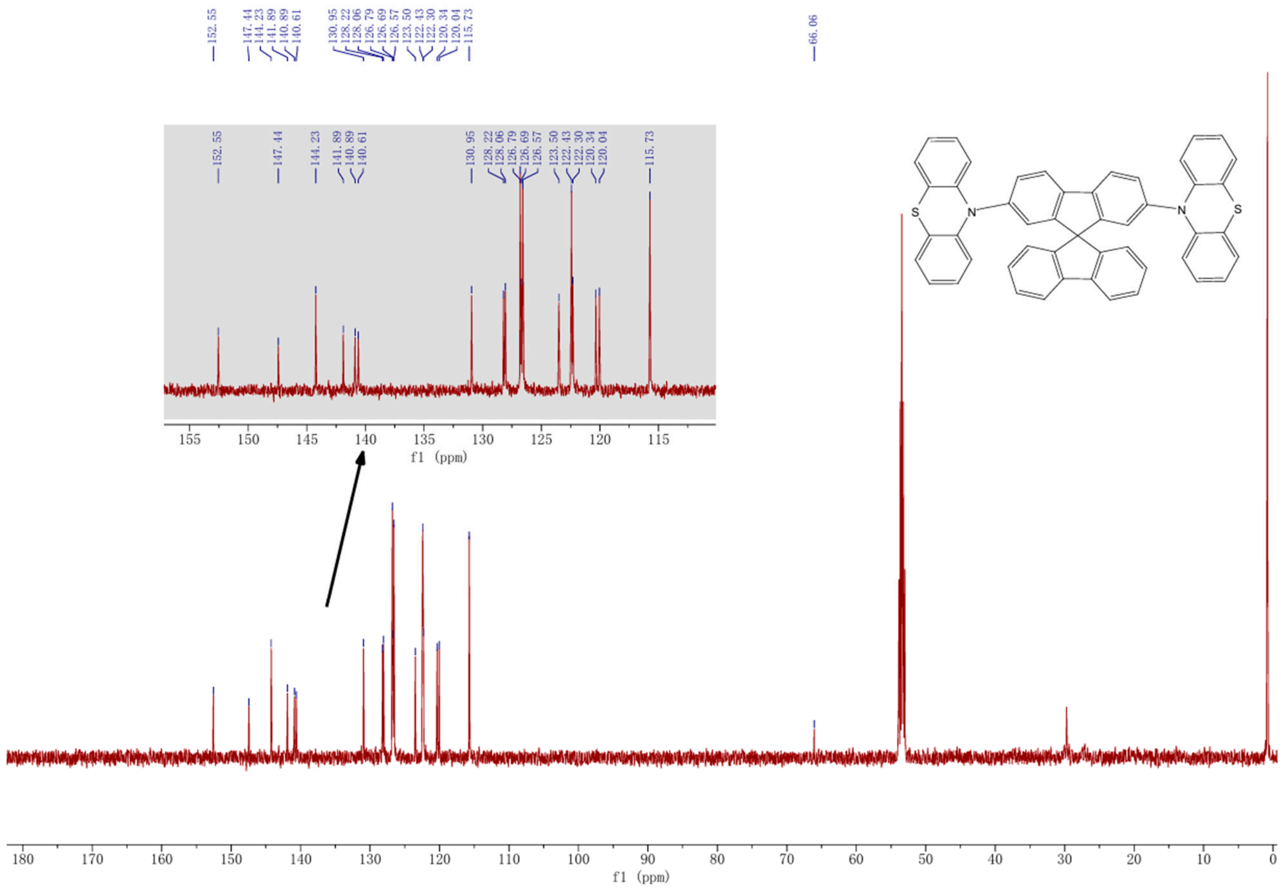

Figure S5. ^{13}C NMR spectrum of compound SRFR-2PTZ in CD_2Cl_2.

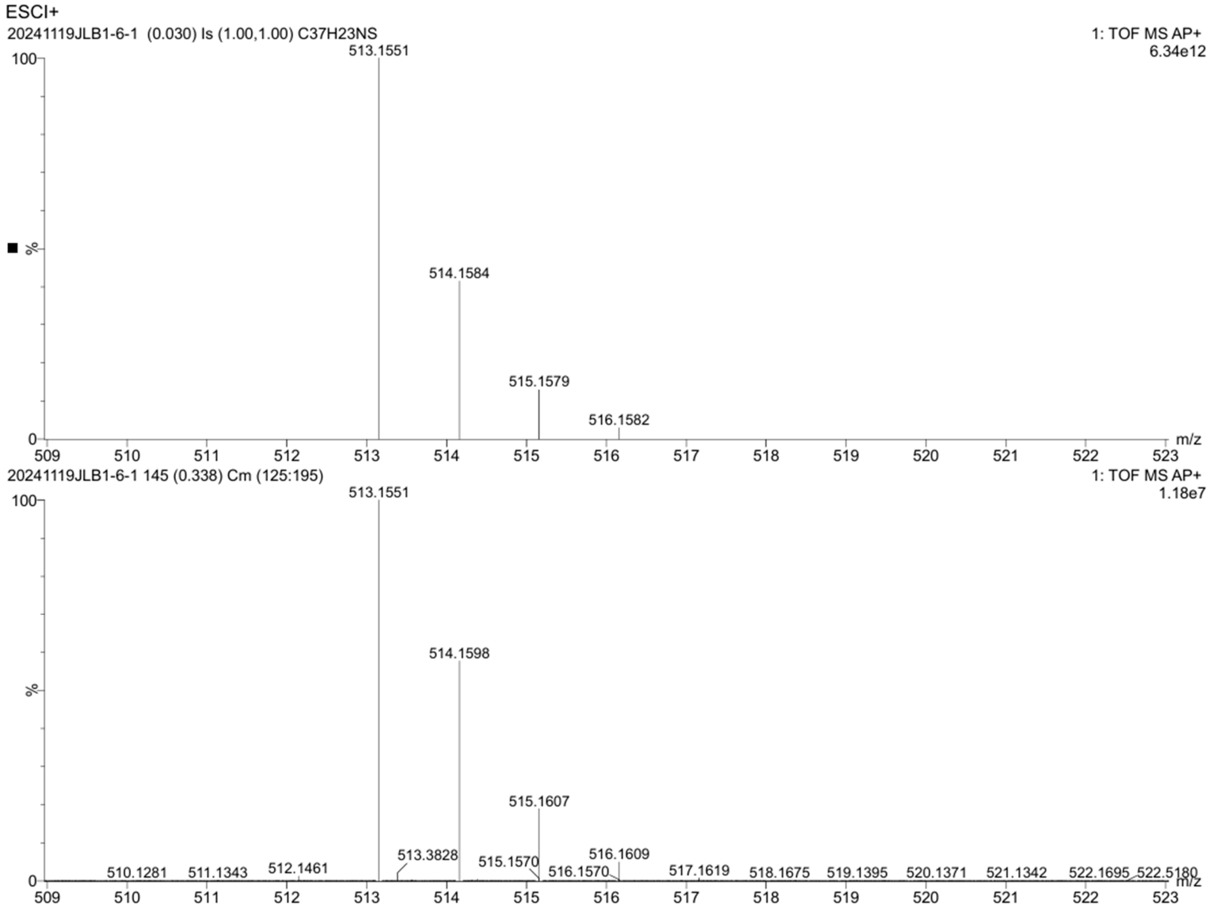

Figure S6. HR-MS spectrum of compound SRFR-1PTZ.

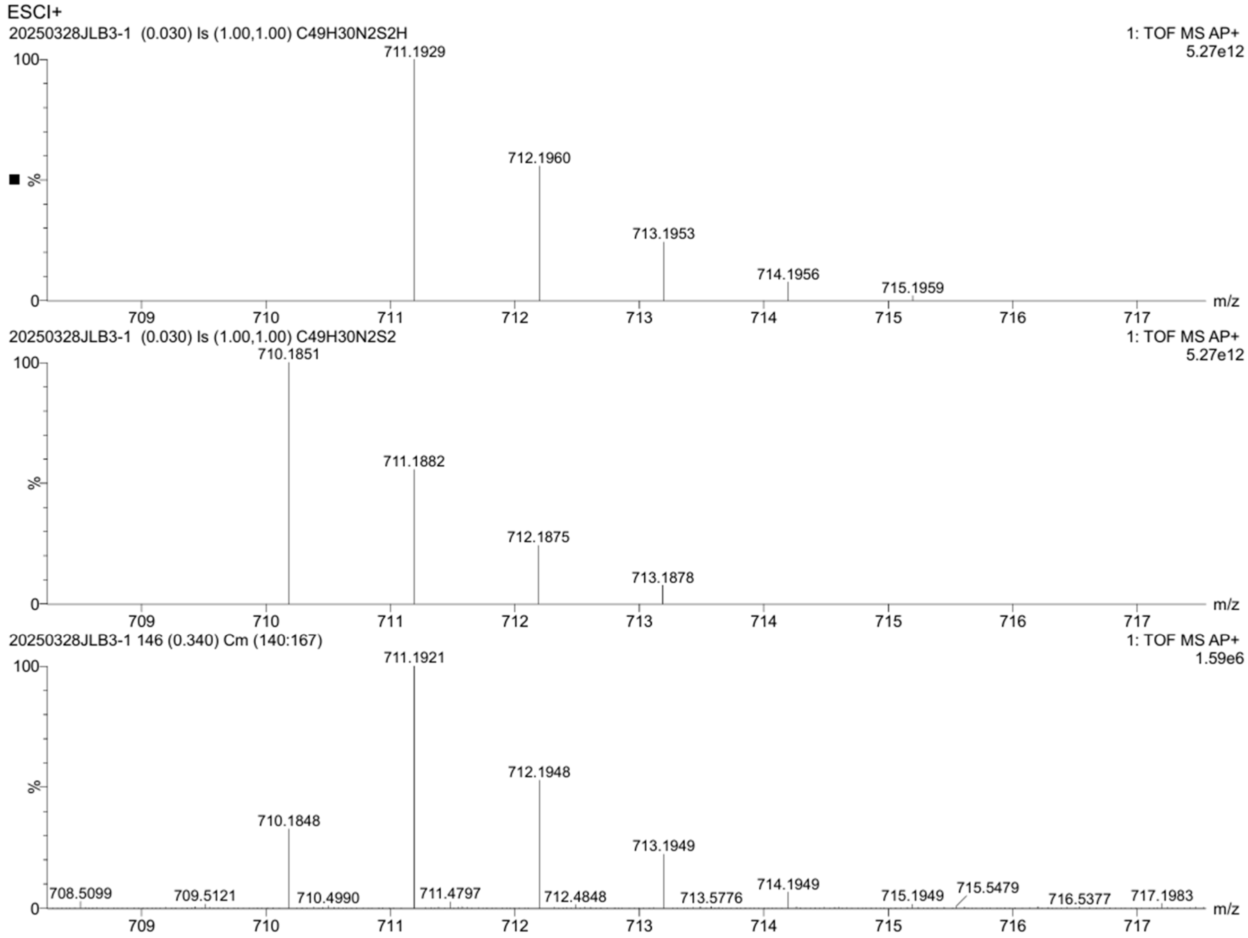

Figure S7. HR-MS spectrum of compound SRFR-2PTZ.

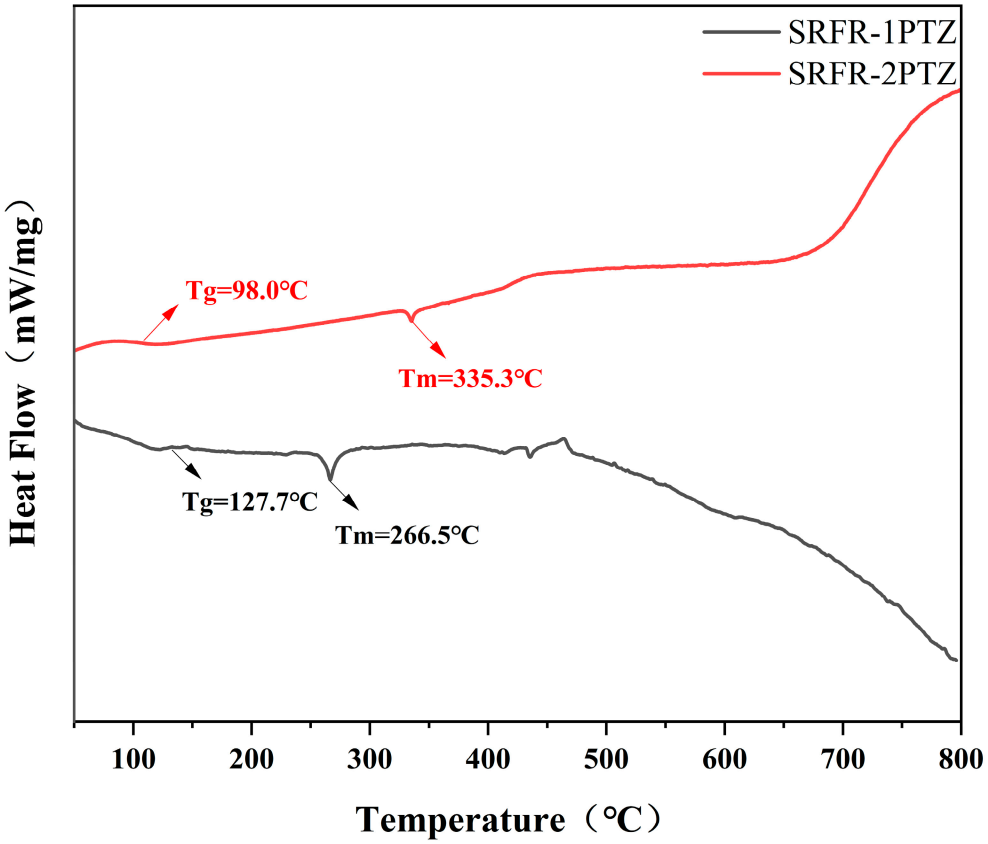

Figure S8. DSC of SRFR-1PTZ and SRFR-2PTZ recorded under nitrogen atmosphere. It could be seen from the figure that the glass transition temperature (T_g) of SRFR-1PTZ was 127.7 °C and the melting temperature (T_m) was 266.5 °C, while the glass transition temperature (T_g) of SRFR-2PTZ was 98.0 ℃ and the melting temperature (T_m) was 335.3 ℃.

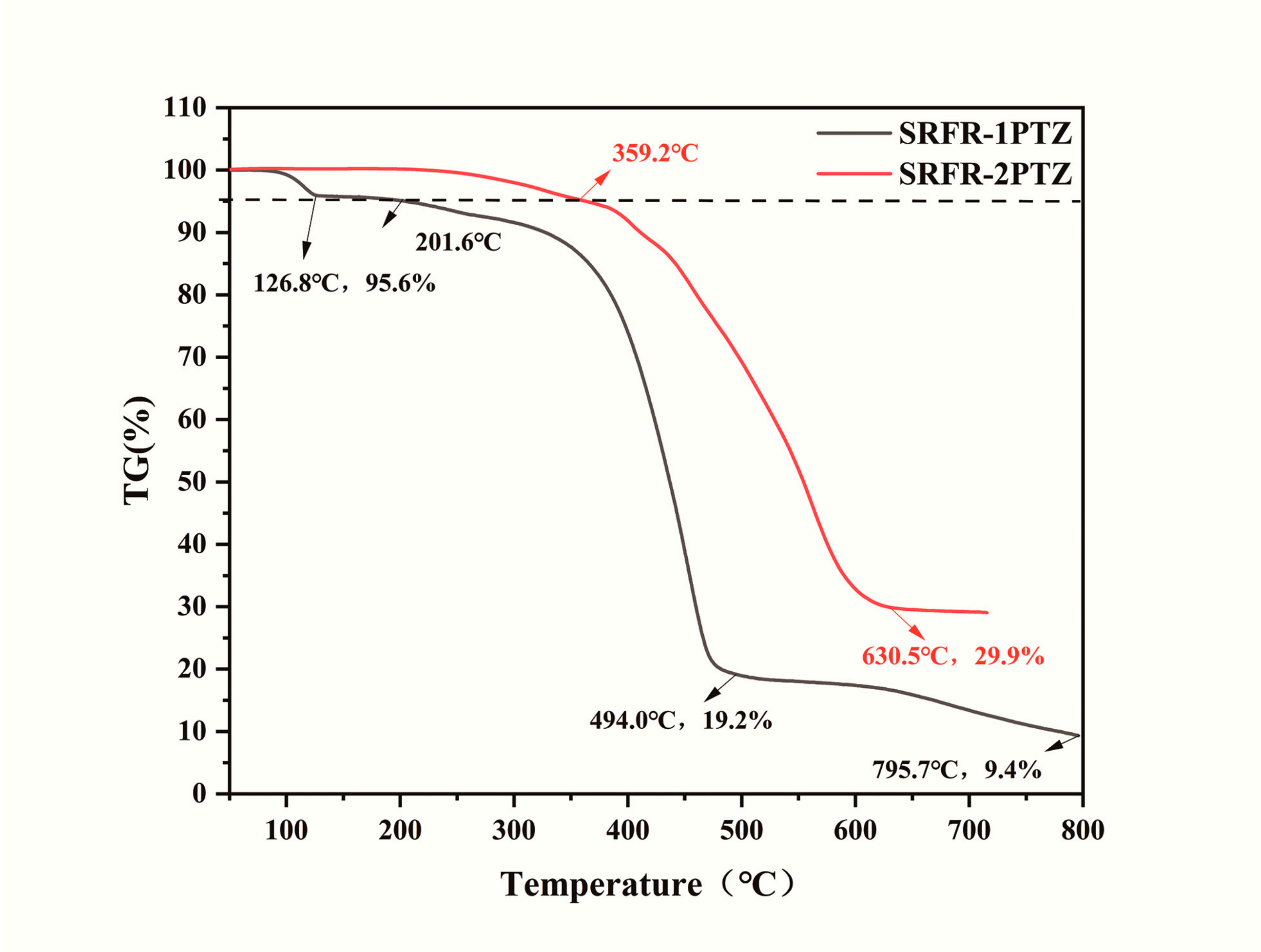

Figure S9. TGA of SRFR-1PTZ and SRFR-2PTZ recorded under nitrogen atmosphere. SRFR-1PTZ TGA had a weight loss of 5%, that was, the remaining 95% had a temperature of 201.6 ℃. SRFR-2PTZ TGA had a weight loss of 5%, which meant, the remaining 95% had a temperature of 359.2 ℃.

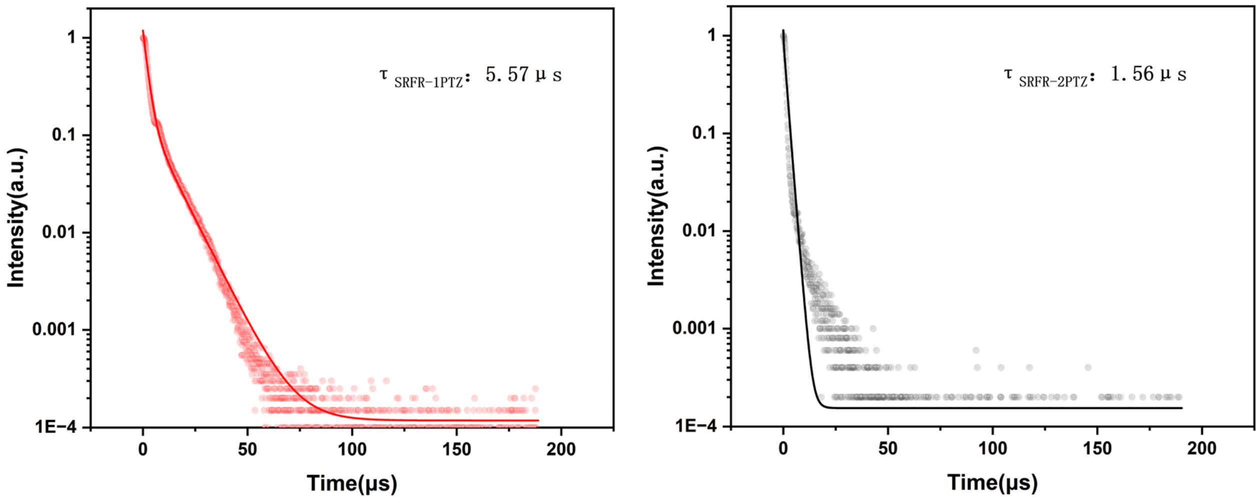

Figure S10. The transient photoluminescence lifetimes of SRFR-1PTZ and SRFR-2PTZ.

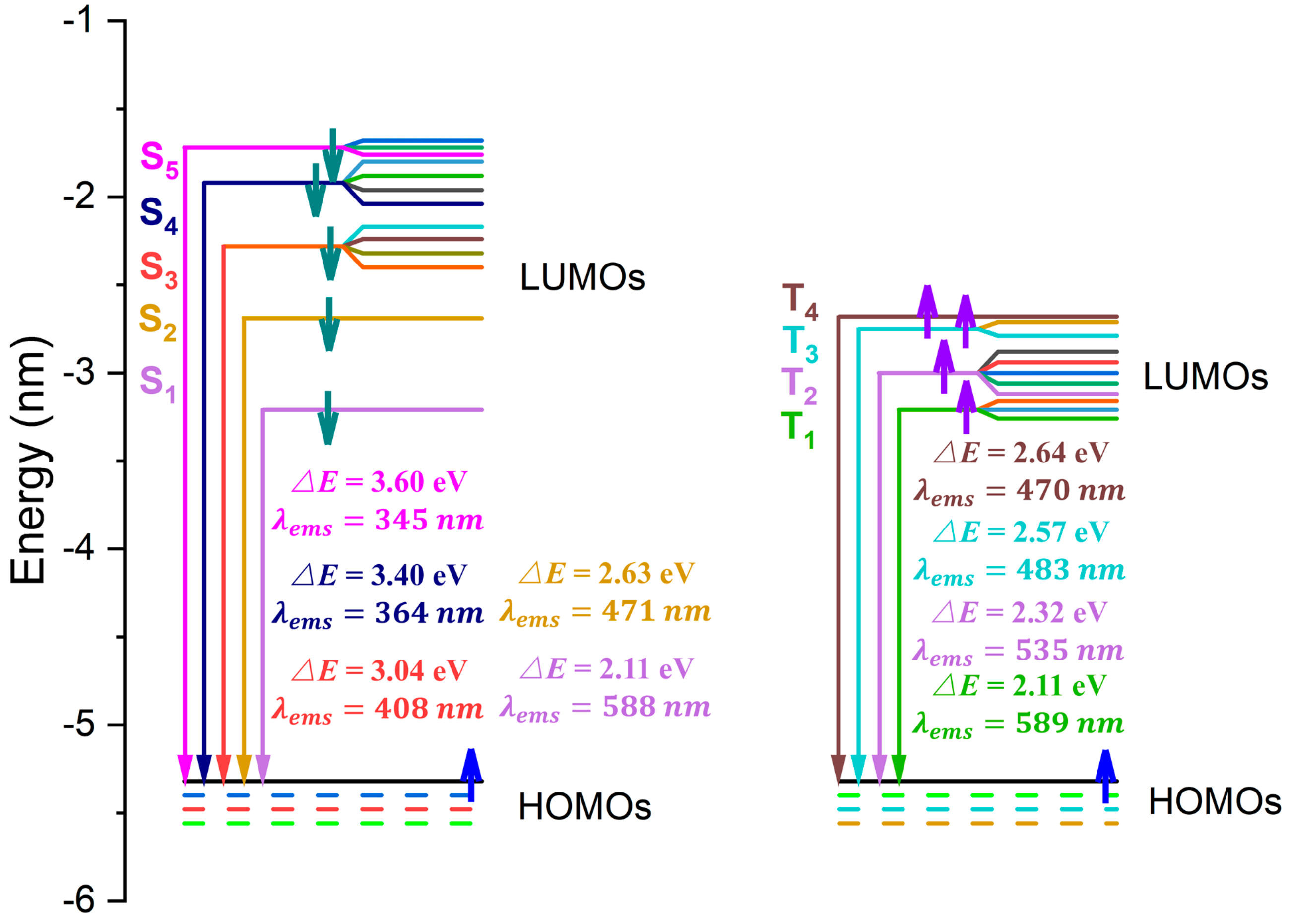

Figure S11. Presentation of the energy levels and the energy gaps for SRFR-2PTZ under DFT and TDDFT calculations.

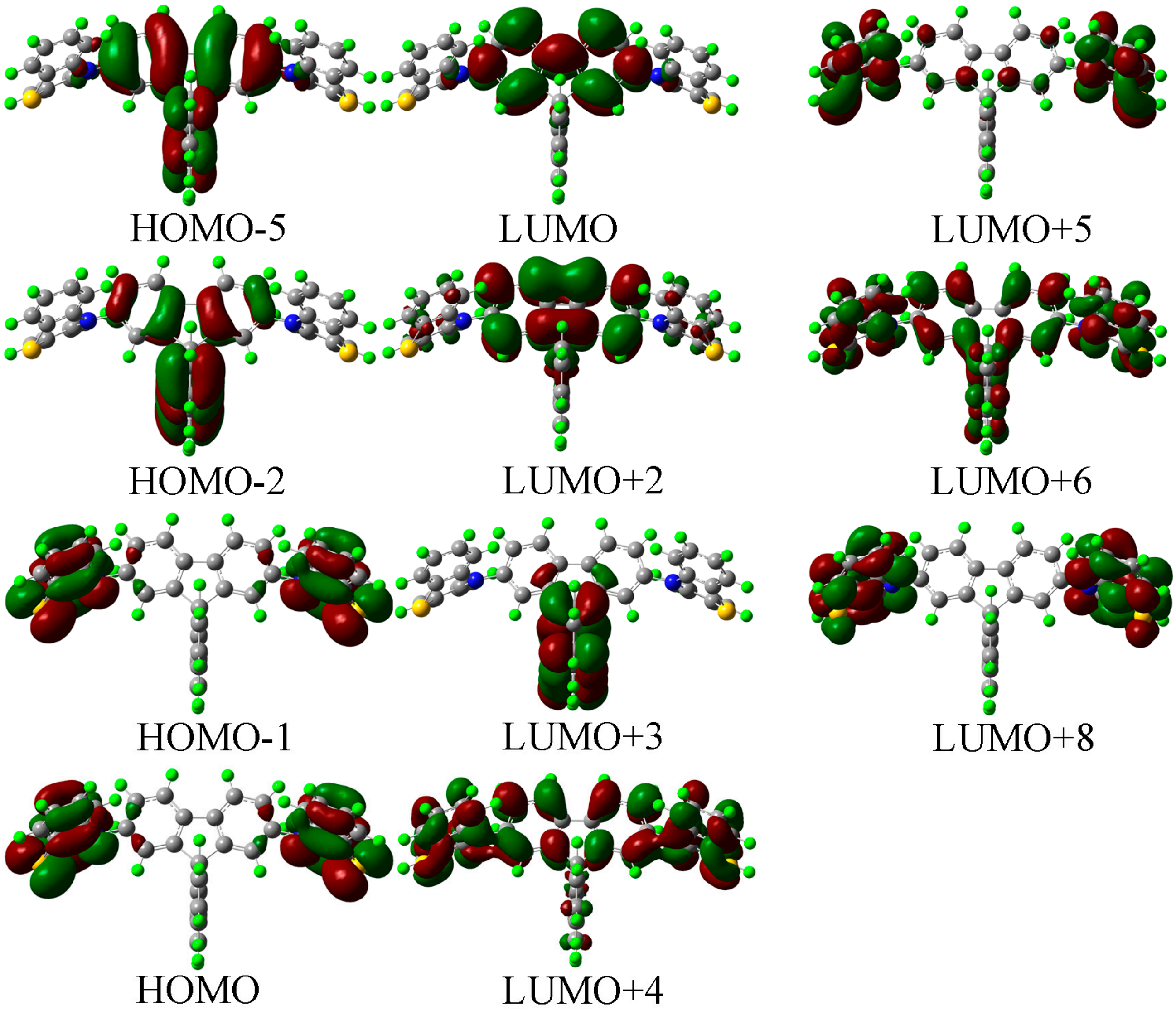

Figure S12. Presentation of the orbital composition distribution of the activation orbitals joined in charge-transfer transitions for SRFR-2PTZ under DFT and TDDFT calculations.

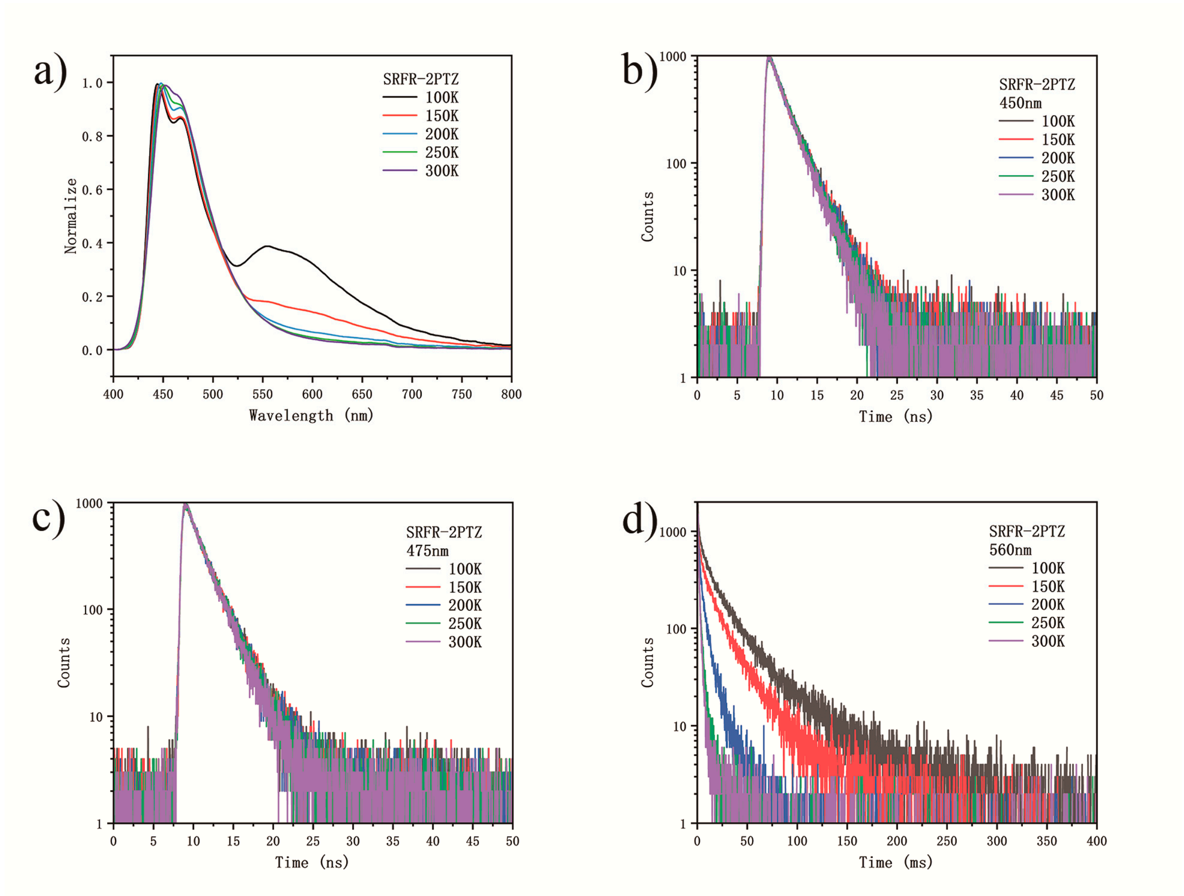

Figure S13. a) Temperature-dependent luminescence spectra of SRFR-2PTZ as solid from 100 to 300 K. b), c) and d) Temperature-dependent transient PL decay curves of SRFR-2PTZ with 450 nm, 475 nm and 560 nm.

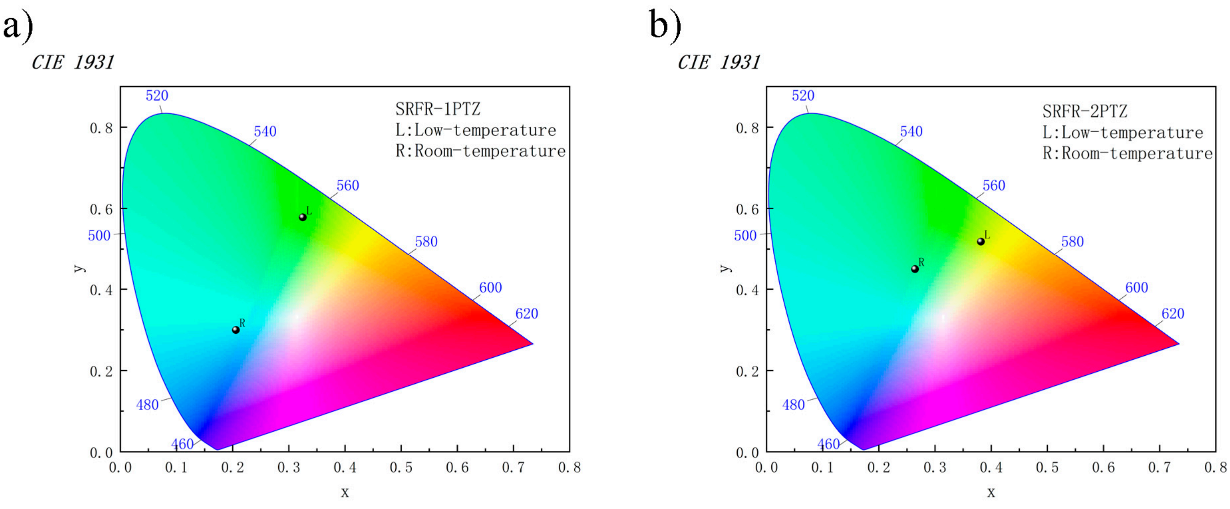

Figure S14. Spectral color coordinates of SRFR-1PTZ a) and SRFR-2PTZ b) complexes at room and low temperatures.

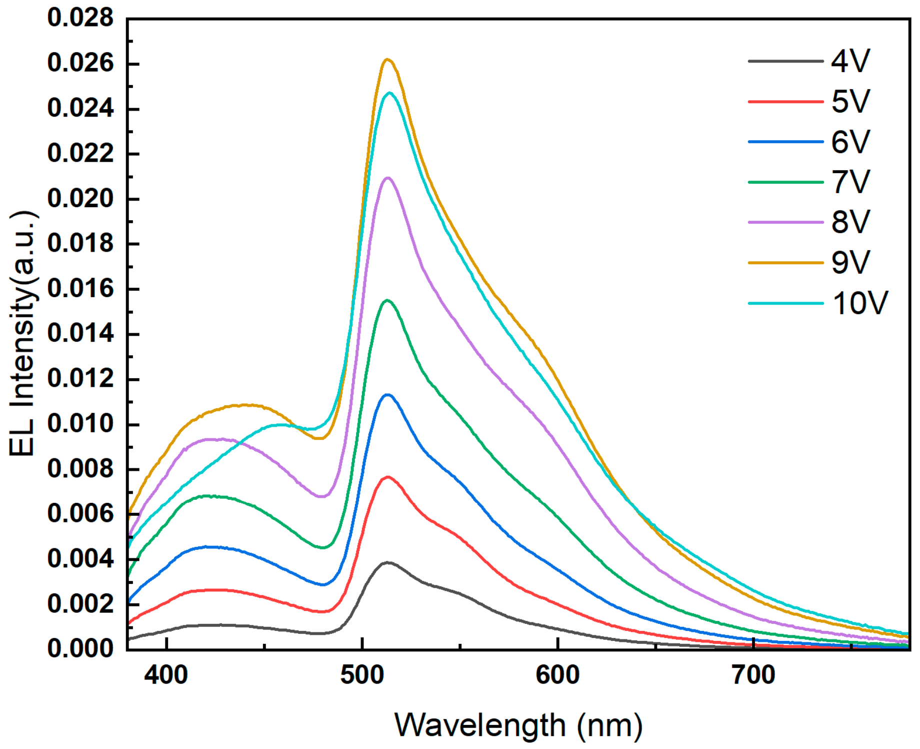

Figure S15. EL spectra under the driving voltages from 4 V to 10 V (Raw) for SRFR-2PTZ.

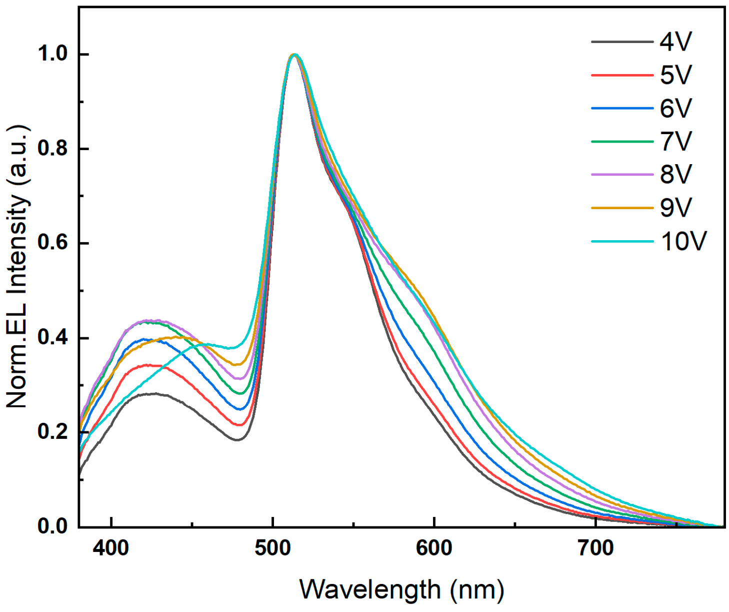

Figure S16. EL spectra under the driving voltages from 4 V to 10 V (Normalized) for SRFR-2PTZ.

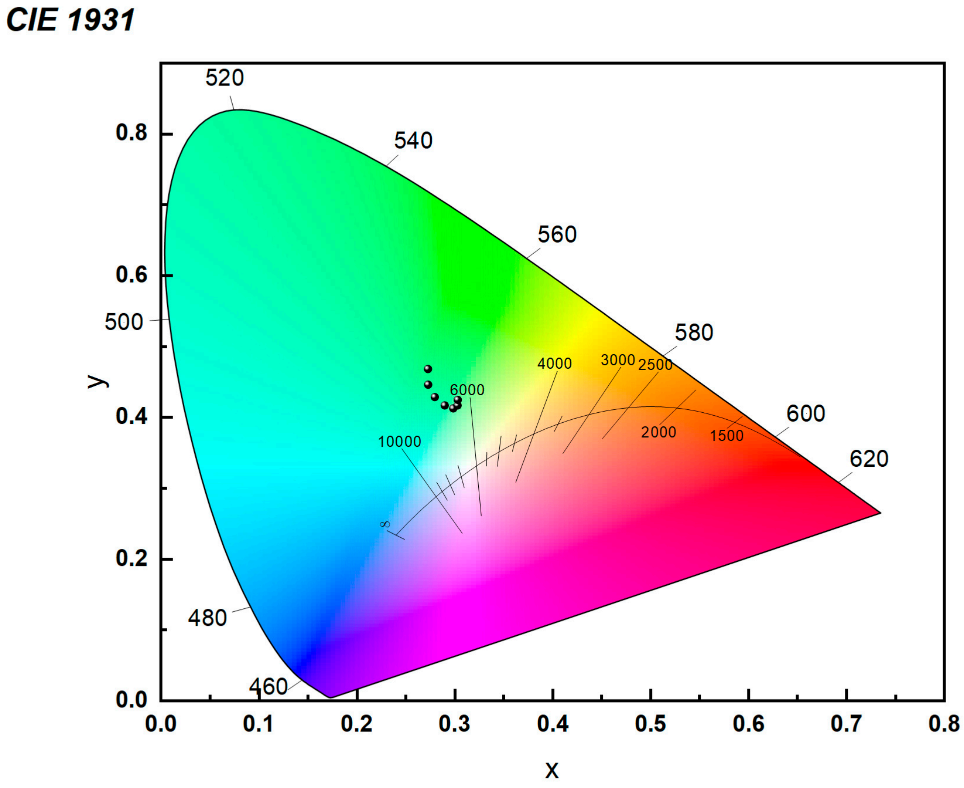

Figure S17. CIE coordinates and color-temperatures measured under the driving voltages from 4 V to 10 V for SRFR-2PTZ.

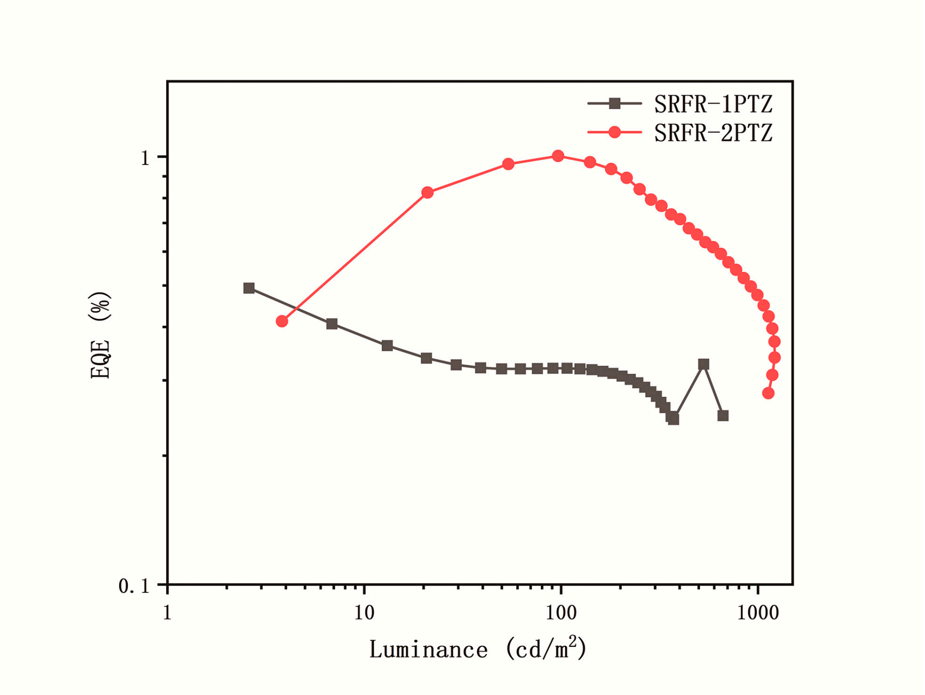

Figure S18. The L-EQE curves of the two compounds.

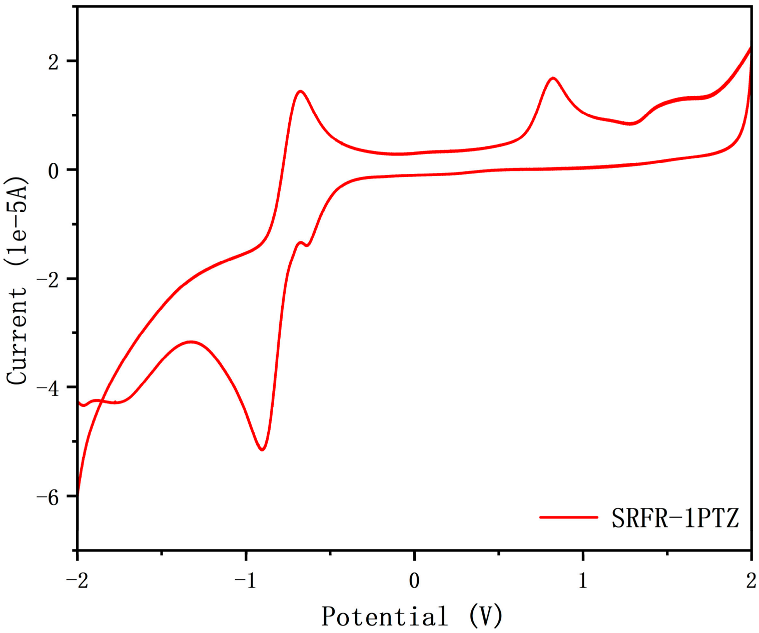

Figure S19. Cyclic voltammetry (CV) measurement of compound SRFR-1PTZ.

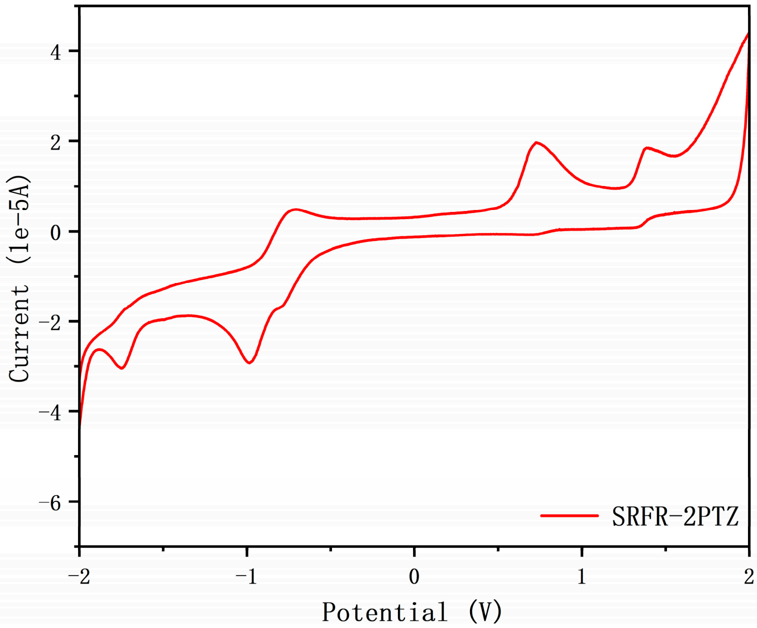

Figure S20. Cyclic voltammetry (CV) measurement of compound SRFR-2PTZ.

4. References:

1. Byeon, S.Y.; Kim, J.H.; Lee, J.Y. CN-modified host materials for improved efficiency and lifetime in blue phosphorescent and thermally activated delayed fluorescent organic light-emitting diodes. *ACS Appl. Mater. Interfaces* **2017**, *9*, 13339-13346. [CrossRef]
2. Choi, S.H.; Lee, C.H.; Adachi, C.; Lee, S.Y. Highly effective nicotinonitrile-derivatives-based thermally activated delayed fluorescence emitter with asymmetric molecular architecture for high-performance organic light-emitting diodes. *Dyes Pigm.* **2020**, *172*, 107849. [CrossRef]
3. Jiang, P.; Miao, J.; Cao, X.; Xia, H.; Pan, K.; Hua, T.; Lv, X.; Huang, Z.; Zou, Y.; Yang, C. Quenching-resistant multiresonance TADF emitter realizes 40% external quantum efficiency in narrowband electroluminescence at high doping level. *Adv. Mater.* **2022**, *34*, 2106954. [CrossRef]
4. Serevičius, T.; Skaisgiris, R.; Kreiza, G.; Dodonova, J.; Kazlauskas, K.; Orentas, E.; Tumkevičius, S.; Juršėnas, S. TADF parameters in the solid state: An easy way to draw wrong conclusions. *J. Phys. Chem. A* **2021**, *125*, 1637-1641. [CrossRef]
5. Baryshnikov, G.; Minaev, B.; Ågren, H. Theory and calculation of the phosphorescence phenomenon. *Chem. Rev.* **2017**, *117*, 6500-6537. [CrossRef]
